# Using Natural Language Processing Techniques to Provide Personalized Educational Materials for Chronic Disease Patients in China: Development and Assessment of a Knowledge-Based Health Recommender System

**DOI:** 10.2196/17642

**Published:** 2020-04-23

**Authors:** Zheyu Wang, Haoce Huang, Liping Cui, Juan Chen, Jiye An, Huilong Duan, Huiqing Ge, Ning Deng

**Affiliations:** 1 Ministry of Education Key Laboratory of Biomedical Engineering College of Biomedical Engineering and Instrument Science Zhejiang University Hangzhou China; 2 Engineering Research Center of Cognitive Healthcare of Zhejiang Province (Sir Run Run Shaw Hospital) Zhejiang University Hangzhou China; 3 General Hospital of Ningxia Medical University Yinchuan China; 4 Sir Run-Run Shaw Hospital Zhejiang University School of Medicine Zhejiang University Hangzhou China

**Keywords:** health education, ontology, natural language processing, chronic disease, recommender system

## Abstract

**Background:**

Health education emerged as an important intervention for improving the awareness and self-management abilities of chronic disease patients. The development of information technologies has changed the form of patient educational materials from traditional paper materials to electronic materials. To date, the amount of patient educational materials on the internet is tremendous, with variable quality, which makes it hard to identify the most valuable materials by individuals lacking medical backgrounds.

**Objective:**

The aim of this study was to develop a health recommender system to provide appropriate educational materials for chronic disease patients in China and evaluate the effect of this system.

**Methods:**

A knowledge-based recommender system was implemented using ontology and several natural language processing (NLP) techniques. The development process was divided into 3 stages. In stage 1, an ontology was constructed to describe patient characteristics contained in the data. In stage 2, an algorithm was designed and implemented to generate recommendations based on the ontology. Patient data and educational materials were mapped to the ontology and converted into vectors of the same length, and then recommendations were generated according to similarity between these vectors. In stage 3, the ontology and algorithm were incorporated into an mHealth system for practical use. Keyword extraction algorithms and pretrained word embeddings were used to preprocess educational materials. Three strategies were proposed to improve the performance of keyword extraction. System evaluation was based on a manually assembled test collection for 50 patients and 100 educational documents. Recommendation performance was assessed using the macro precision of top-ranked documents and the overall mean average precision (MAP).

**Results:**

The constructed ontology contained 40 classes, 31 object properties, 67 data properties, and 32 individuals. A total of 80 SWRL rules were defined to implement the semantic logic of mapping patient original data to the ontology vector space. The recommender system was implemented as a separate Web service connected with patients' smartphones. According to the evaluation results, our system can achieve a macro precision up to 0.970 for the top 1 recommendation and an overall MAP score up to 0.628.

**Conclusions:**

This study demonstrated that a knowledge-based health recommender system has the potential to accurately recommend educational materials to chronic disease patients. Traditional NLP techniques combined with improvement strategies for specific language and domain proved to be effective for improving system performance. One direction for future work is to explore the effect of such systems from the perspective of patients in a practical setting.

## Introduction

### Background

Chronic (or noncommunicable) diseases are the most prevalent and costly conditions worldwide [1]. To improve the survival rate and life quality of chronic disease patients, long-term self-management and supervision and intervention from doctors are essential [2]. However, in practice, some patients don’t perform effective self-management regimes due to the lack of necessary knowledge, skills, and confidence, which results in decreased treatment effectiveness or even treatment failure [3-6]. Health education from health care providers has been considered an important intervention for improving patient awareness and self-management abilities in chronic disease management [7-9].

The development of information technologies has promoted the advent of eHealth-enhanced chronic disease management, which changed the form of patient educational materials from traditional paper materials to electronic materials [10-13]. Patients can either receive expert-vetted materials from their doctors or perform self-learning on the internet. To date, a large amount of patient educational materials exist on the internet; however, the quality of health information in these materials is highly variable [14-17]. Patients without a medical background may find it hard to identify the most relevant and valuable materials for themselves [18,19]. A system that is capable of automatically identifying and recommending appropriate materials to patients based on their needs [20] or preferences [21] would be applicable to solve the above problems. Such a system can be categorized as a kind of health recommender system (HRS).

As one of the specializations of recommender systems, an HRS aims to recommend relevant medical information to health professionals or patients [22]. A number of works regarding the design and implementation of HRSs have been published, providing recommendations in different areas such as diets [23], health care services [24], educational materials [25], and decision-making advice for doctors [26]. Pincay et al [27] summarized HRSs into 4 recommendation areas: wellness, diagnosis and medication, health care services, and medical resources. Among these areas, patient educational materials belong to the medical resources. Given the fact that only 3% of the articles focused on this area [27], in this study we aimed to develop an HRS to provide personalized educational materials for patients with chronic diseases.

### Related Work

In a health context, multiple methods from the computer science field have been applied to compute relevant recommendations. According to a review [22], two main approaches were used for HRSs. One is the information retrieval (IR) approach, in which the recommendations are generated based on a query that describes the user’s information interest. Another approach is the recommendation algorithm (RA) approach, which has been widely used in the context of online shopping and advertisement [28]. Unlike the IR approach that returns relevant results matching the user query, the RA approach generates personalized results tailored to the users’ potential needs or preferences.

Among different RA approaches [29], the most applied methods in HRSs are collaborative filtering, content-based, and knowledge-based methods [27]. The collaborative filtering method recommends to the active user the items that other users with similar preferences liked in the past [30]. One major drawback of collaborative filtering is the cold-start problem, referring to the problem that a new user who has not rated any items cannot receive recommendations (called new user problem) or a new item with too few ratings cannot be recommended (called new item problem). Compared with collaborative filtering, the content-based method solves the new item problem by recommending items with content-similar features as the user liked in the past. The similarity of items is calculated based on the features associated with the compared items [31]. The knowledge-based method can be viewed as an extension of the content-based method, by considering how items meet user preferences or needs based on domain knowledge, instead of user ratings [32]. Ontologies are often used for knowledge representation in the knowledge-based method [33].

Compared with collaborative filtering and the traditional content-based method, the knowledge-based method is considered more appropriate in the context of e-learning. In e-learning environments, different learners have different characteristics such as background knowledge, learning history, and competence level; therefore, even if two learners have similar ratings, they will require different recommendations if their characteristics are not the same [34]. Conventional RAs such as collaborative filtering and content-based methods recommend items to users based solely on ratings, while the knowledge-based method can personalize user profiles to match the user characteristics through knowledge models such as ontologies [35]. The aggregation of domain knowledge about the learner and learning resources has been proven to improve the quality of recommendations, meanwhile alleviating other conventional drawbacks such as cold-start and rating sparsity problems [36]. Since patient self-learning based on electronic materials can be considered as a kind of e-learning, a knowledge-based HRS may be a better choice to incorporate additional information about patients for recommendation.

Several studies have explored the feasibility of an HRS for recommending patient educational materials. Kandula et al [20] used the IR approach to recommend relevant educational materials to diabetic patients. They applied the topic modeling method (latent Dirichlet allocation) to identify and match topics between educational materials and patients’ electronic medical records. Zeng et al [37] also adopted the IR approach to recommend educational materials for diabetic patients. Instead of inferring patients’ needs from electronic medical record notes, they constructed patients’ questions on the forum as a query and then compared two algorithms (latent Dirichlet allocation and semantic group). Sanchez et al [25] built a content-based recommender system that links patients to reputable health educational websites from MedlinePlus for a given health video from YouTube. They used the BioPortal application programming interface (API) to extract Systematized Nomenclature of Medicine–Clinical Terms (SNOMED-CT) terms from videos viewed by patients, and then used the MedlinePlus API to provide relevant MedlinePlus recommendations based on these terms. In their subsequent work [38], they introduced natural language processing (NLP) techniques to extract SNOMED-CT terms from video content, and then added the Bio-ontology API to improve the results for obtaining synonymous MedlinePlus terms. Wang et al [21] implemented a cloud-based mobile health information recommendation system that included a collaborative recommender and a physiological indicator-based recommender. These studies proved that HRSs have the potential to provide personalized education for patients using different information technologies. However, to the best of our knowledge, no studies to date have formally concentrated on a knowledge-based HRS for chronic disease patient education. Moreover, most of the materials are in English; no studies have provided the feasibility evidence of recommending materials in Chinese.

### Objectives

Here we propose a knowledge-based HRS that recommends relevant educational materials to chronic disease patients according to their health data. The materials are limited to Chinese documents, and several NLP techniques will be used to preprocess the text-based materials. Further, this study explores the effect of the system through a pilot evaluation based on a manually annotated test collection.

## Methods

### Study Design

In this study, we had a corpus of patient educational materials retrieved from multiple sources and a data set of patients collected from a telehealth system. The task of this study was to design and implement an automated recommender system that can discover patients’ potential needs from their health data, and then recommend the most relevant educational materials to them. In addition, we needed to design an assessment method to evaluate system performance.

**Figure 1:**
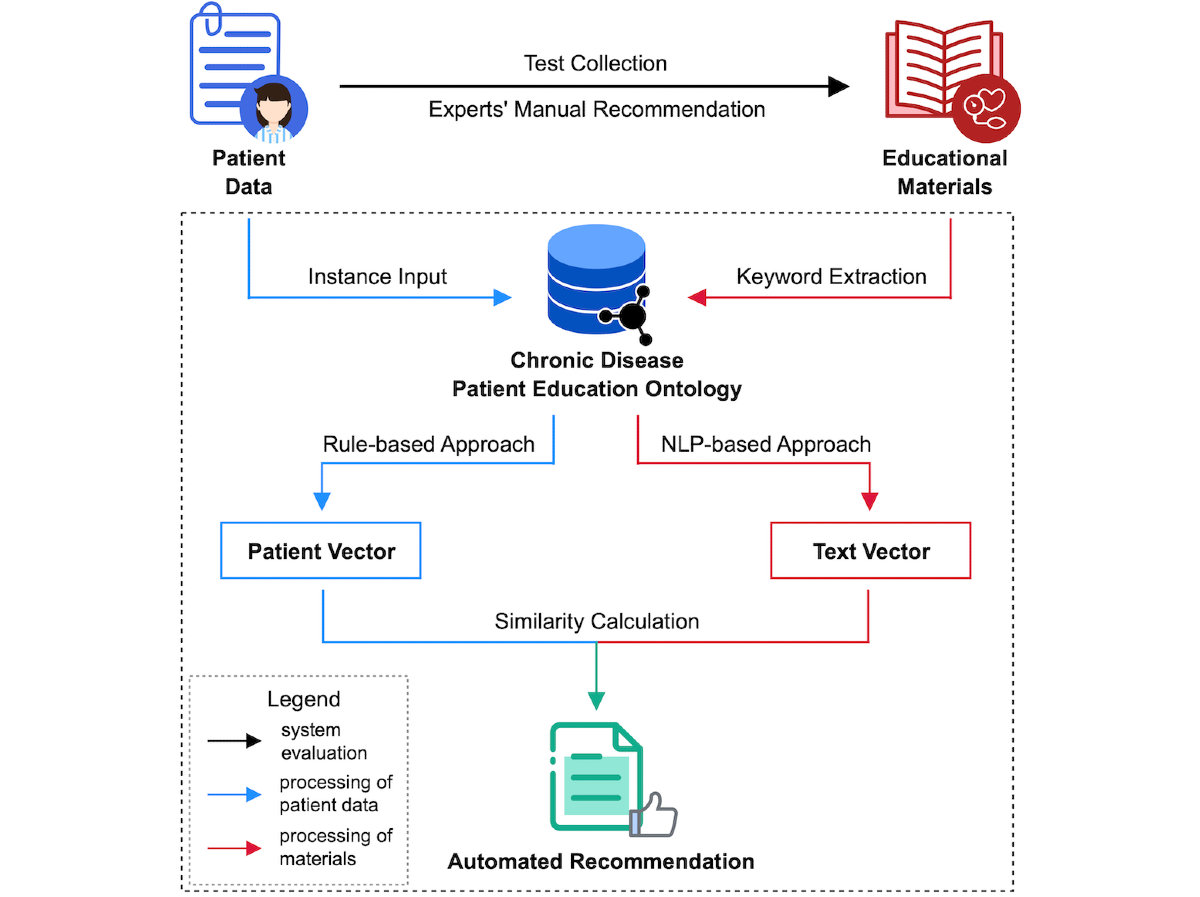
Overall study design.

The study was designed based on these tasks. Figure 1 illustrates the overall study design. The complete recommendation process is presented in the dotted box. The core of the recommendation process is a custom ontology called Chronic Disease Patient Education Ontology (CDPEO), which describes patient characteristics for recommendation generation. Patient data and educational materials will be converted to vectors through CDPEO. Patient vectors and text vectors will have the same length, and the final recommendation results will be generated based on the similarity between these vectors. Patient data will be converted through a rule-based approach (blue arrows in Figure 1), while educational materials will be converted through an NLP-based approach (red arrows in Figure 1). System evaluation will be conducted based on a test collection of educational materials manually assembled by domain experts (black arrows in Figure 1).

### Data Collection

Patient educational materials used in this study came from multiple sources including websites, guidelines, and books, which have been reviewed and approved by several physicians (see Multimedia Appendix 1 for further information). We retrieved 88,746 documents in Chinese from these sources. Among these documents, 511 were manually extracted in the form of plain text from the guidelines or books, while the others were crawled from the websites and transformed into plain text using a Python software library called Beautiful Soup. Patient data used in this study came from a telehealth system, which is a pathway-driven mobile health (mHealth) system for chronic disease management. The system aims to provide comprehensive self-management support for patients and executable intervention plans for care providers [39,40]. Currently, more than 5000 patients are using this system in Ningxia and Zhejiang provinces. We randomly selected 50 patients and collected their data to develop and test our recommender system. Data included demographics, laboratory test results, disease histories, self-monitoring records, and questionnaire results.

### Informed Consent and Ethical Consideration

Patients registered in the telehealth system have signed inform consent forms for accessing and using their privacy data. The domain experts signed informed consent forms as well. All procedures were performed in accordance with the ethical guidelines for biomedical research involving human subjects at Ningxia Medical University.

### System Development Steps

#### Overview

The development process of the system can be divided into 3 stages. In stage 1, we constructed an ontology (CDPEO) for patient education mainly based on the collected data and materials. In stage 2, we designed and implemented an algorithm to generate the recommendations based on the ontology. In stage 3, we integrated the ontology and the algorithm into our mHealth system for practical use.

#### Stage 1: Ontology Construction

**Figure 2:**
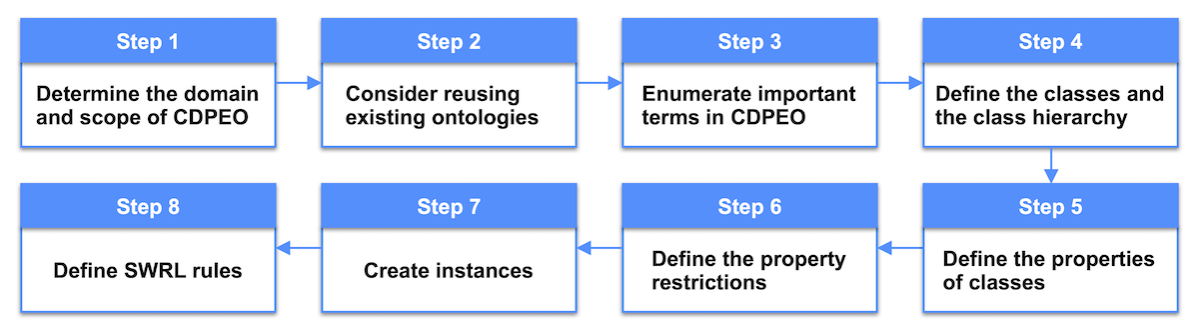
Chronic Disease Patient Education Ontology construction steps.

The construction of CDPEO followed a widely used ontology engineering methodology [41], as shown in Figure 2. First, we defined the domain and scope of CDPEO by sketching a list of questions the ontology should be able to answer. This method is called competency questions [42]. Through this step, we confirmed that CDPEO will be used as a reference model for the representation of patient data and educational materials, and the intended output of CDPEO is a comprehensive label set for patient education. Second, we searched for reusable existing ontologies on BioPortal (a Web repository of biomedical ontologies) using keywords “hypertension,” “diabetes,” “chronic disease,” and “patient education.” A total of 9 ontologies were screened. However, due to the specific domain of our ontology, classes and properties defined in the existing ontologies could hardly be refined for our particular task. Therefore, we created CDPEO from scratch. Third, we collected all terminologies that might be used in the ontology. These terms were mainly collected from educational materials and patient data records. We selected terms able to describe patient characteristics or material topics, as well as concepts that might be involved in the recommendation process. All terms were originally in Chinese, translated into English for ontology construction. After applying this step, we obtained a relatively comprehensive term list with 54 terms. The term list was reviewed by the physicians as well. The detailed outputs of these 3 steps can be found in Multimedia Appendix 2.

Fourth, based on the term list, we defined the classes and the class hierarchy of CDPEO through a top-down approach, which started with defining the most general concepts in the domain and subsequently specializing the concepts. CDPEO was built in two main levels of abstraction. Level 1 included 5 terms (demographic, disease, physiological index, lifestyle, and medication) that described characteristics contained in patient data. Level 2 included the detailed elements for each of the level 1 classes. Fifth, we defined the properties of classes based on the remaining terms to describe the internal structure of concepts. The properties consisted of two types: object properties and data properties. Object properties are relations between two individuals (ie, instances of classes), while data properties describe relations between an individual and a data value.

Sixth, we defined property restrictions to complete the precise semantics of the classes. These restrictions were represented as a set of axioms including property and individual axioms. Property axioms described the facets of properties such as value type, number of values, and domain and scope of properties. Individual axioms described anonymous classes of individuals based on the relations that members of the class participate in. Seventh, we created individual instances of classes in the hierarchy. CDPEO was instantiated by the patient data. We defined a class called patient profile in the top level to be the core component of the instances. The characteristic instances were created and bound to the patient profile instance. Finally, we used the Semantic Web Rule Language (SWRL) [43] to encode rules for complex inferences, for example, generating a new property of an instance. SWRL is based on rule markup language and compatible with the W3C Web Ontology Language (OWL) [43]. In CDPEO, the SWRL rules were defined to evaluate the patient data and generate a fixed-length vector (33-dimensional) for recommendation generation.

#### Stage 2: Recommendation Generation

**Figure 3:**
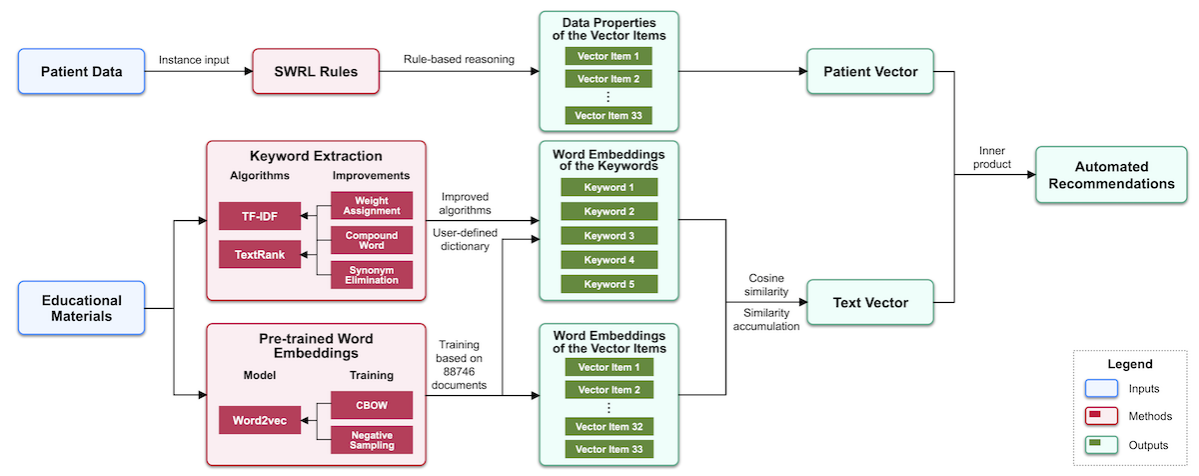
Recommendation generation steps.

Based on the constructed ontology, we designed and implemented an algorithm to automatically generate recommendations of educational materials given patient data. The core idea of the algorithm was mapping patient data and educational materials to an identical vector space. The vector space came from the ontology, containing 33 terms that can describe patient data characteristics and document topics. The complete recommendation generation steps are shown in Figure 3.

For patient data, we used SWRL rules to infer the item values of the vector. The values were in the range of 0 to 3, which indicated the severity of the corresponding term. For example, in the vector space existed a term called blood pressure (BP), whose value was inferred based on the latest self-monitoring record of the patient. If the BP record was below 140/90 mm Hg, then the item value would be 0, otherwise the value would be 1, 2, or 3 based on the severity of the BP record (3 means the worst). All reasoning procedures were completed by the SWRL rules, and the results were saved as data properties of the corresponding patient profile instance.

For educational materials, we applied an NLP-based approach to map documents to the vector space. First, we summarized the topic of each document by keywords. In this study, we used 2 famous statistical algorithms, term frequency–inverse document frequency (TF-IDF) [44] and TextRank [45], to extract keywords from educational materials. In TF-IDF, the IDF scores were calculated from the educational material corpus; in TextRank, undirected graphs for a co-occurrence window of 2 were used. Five keywords were extracted for each document. Furthermore, three strategies were introduced to improve extraction performance specifically for Chinese educational materials: weight assignment, compound word identification, and synonym elimination. Table 1 summarizes these strategies, with a description of each strategy and its effects. A simple example of each strategy for intuitive interpretation can be found in Multimedia Appendix 3.

In weight assignment, we set an additional weight value for some words based on the observation of the corpus. We observed that for patient educational materials in Chinese, title words and nouns were more likely to be the keywords while verbs were less likely to be the keywords. When performing keyword extraction, a weight greater than 1 could improve the likelihood of being the keyword while a weight less than 1 could reduce the likelihood. Consequently, weights of 3, 1.2, and 0.8 were assigned to title words, nouns, and verbs, respectively, by the investigators based on multiple experiments.

In compound word identification, we aimed to identify compound words in patient educational materials. For Chinese documents, sentences need to be segmented into pieces of words, since all words are organized together without blanks in Chinese sentences. We observed that for patient educational materials in Chinese, a compound word was often segmented into separate atom words by the word segmentation algorithm. However, a compound word usually contains more information than a single atom word, and thus is more appropriate for being the keyword. To solve this problem, we designed several filter conditions to identify all compound words in educational materials before word segmentation, and then generated a user-defined dictionary of compound words to customize word segmentation. The filter conditions included co-occurrence frequency, part-of-speech tag for each atom word, and arrangement of atom words.

In synonym elimination, we aimed to eliminate synonyms in the extracted keywords. Synonyms here refer to words composed with similar Chinese characters. We noticed that after introducing compound word identification, synonyms appeared more frequently in keyword extraction. To eliminate these synonyms, we converted each keyword candidate into a one-hot vector based on its character composition. The cosine similarity between each keyword was then calculated to determine if these keywords belong to synonyms. For the identified synonym pair, the longer one was retained while the shorter one was eliminated, since in Chinese longer synonyms usually contain the information in shorter synonyms.

**Figure 4:**
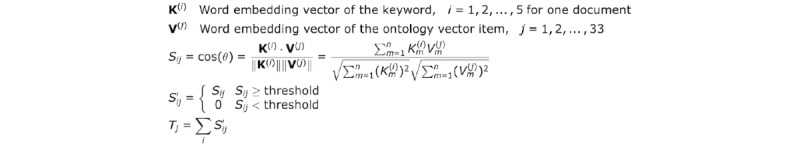
Concrete calculation process of the text vector.

Second, the extracted keywords were mapped to the ontology vector space to generate the text vector based on cosine similarity between keywords and vector items. Similarity was calculated based on a pretrained word embedding of each keyword and vector item. In this study, we used the classic Word2Vec model to obtain statistic embedding vectors for each word [46,47]. The model architecture used was the continuous bag-of-words architecture with a window size of 5, and the training algorithm was the negative sampling method. The training corpus was the collected 88,746 documents concerned with patient education. The item value of the text vector was calculated by the sum of a subset of similarity values between the corresponding item and all keywords. Figure 4 shows the concrete calculation process, in which *T_j_* corresponds to the *j*-th item of the text vector, *n* corresponds to the dimension of the pretrained word embeddings (in this study, *n*=200), threshold corresponds to a value between 0 and 1 (in this study, threshold=0.5).

**Figure 5:**
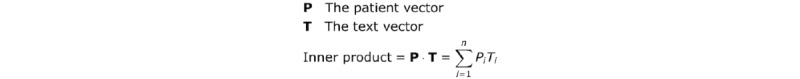
Inner product of the patient vector and text vector.

Given the patient vectors and text vectors, we calculated the inner product of each vector pair to indicate the correlation between patient data and educational materials. The inner product can be interpreted as a nonnormalized cosine similarity that considers the similarity of vectors in both direction and magnitude, as shown in Figure 5, where *n* corresponds to the dimension of the vector (in this study, *n*=33). Larger inner products indicate stronger correlation. Recommendations for a specific patient were generated based on the inner products between the corresponding patient vector and text vectors.

**Table 1:** Summary of the three strategies for improving keyword extraction performance.

Strategy	Description	Effect
Weight assignment	Assign weight of 3, 1.2, and 0.8 to title words, nouns, and verbs, respectively, when performing keyword extraction.	Nouns and title words will be more likely to be keywords, and verbs are less likely to be keywords.
Compound word identification	Use several filter conditions to generate user-defined dictionary of compound words in educational materials for word segmentation.	Compound words that meet filter conditions will be identified and are more likely to be the keywords than atom words.
Synonym elimination	Remove shorter keywords with similar Chinese characters based on cosine similarity between their character compositions.	Two or more keyword candidates with similar character composition will be merged into one keyword to avoid redundancy.

#### Stage 3: mHealth Implementation

In this stage, we incorporated the recommender system (including the ontology and the algorithm) into our mHealth system for practical use. The entire recommender system was implemented as a Web service connected with the mobile app. For each patient, the service will calculate the specific patient vector and text vectors of documents that have not been provided to the patient, and then calculate the inner products between the patient and text vectors. For the recommendation, to reduce computation, we adopted a thresholding method: if the inner product is greater than a certain threshold, then the corresponding document will be considered to be relevant to the patient. The relevant documents will be added to a recommendation queue, pushed to the patient’s smartphone regularly. In addition, one other thing to note is that documents prohibited for reproduction will only be used for training and not be provided to patients.

### Development Tools

Development and evaluation of the system were performed on an iMac (21.5-inch) with an Intel Core i7-5775R CPU 3.3 GHz, with 16 GB main memory running on macOS Mojave 10.14.6. We used the Protégé 5.5.0 open source ontology editor to develop the ontology in OWL2 standard format. The Pellet reasoner was used to enable SWRL reasoning under Protégé. The algorithm for recommendation generation was implemented using Python 3.6 (for source code see Wang and Huang [48]). Several Python libraries have been imported to process the materials: for material retrieval, the Beautiful Soup library (version 4.4.0) was adopted to pull data out of HTML files and transform it into plain text; for keyword extraction, the Jieba library (version 0.39) was adopted for Chinese text segmentation; and for pretrained word embeddings, the Gensim library (version 3.8.1) [49] was adopted to train the Word2Vec model. The Web service was developed under the Flask framework (a lightweight Web app framework for Python), in which the OWLready2 library (version 0.23) [50] was used to manipulate the OWL2 ontology. System evaluation was conducted using Python 3.6.

### System Evaluation

#### Test Collection Assembly

To evaluate system performance, we invited 2 domain experts to assist in assembling a test collection of educational materials. These domain experts are case managers from the General Hospital of Ningxia Medical University. Their daily work is conducting follow-ups on chronic disease patients and providing health education for these patients. Considering the time cost of manual annotation, based on a study in this field [37], 100 educational documents were randomly selected from the corpus to compose the test collection. The system performance evaluation was divided into two parts: evaluation of keyword extraction performance and evaluation of recommendation performance.

#### Evaluation of Keyword Extraction Performance

**Figure 6:**
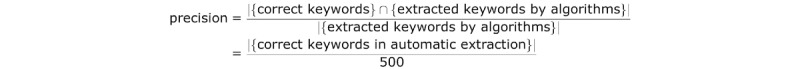
Evaluation metrics of keyword extraction performance.

We asked one expert to extract 5 keywords from each document in the test collection (the other expert reviewed the results). The keywords must have explicitly appeared in the text. We then compared the automatically extracted keywords by the algorithms with the manual extraction results. The evaluation metric was the precision of automatic extraction for the entire test collection, inspired by the evaluation method of TextRank [45], as shown in Figure 6. In this study, since the extracted word counts of manual annotation and algorithms are identical, precision equals recall—the fraction of correctly extracted keywords by algorithms out of the total correct keywords (N=500).

#### Evaluation of Recommendation Performance

**Figure 7:**
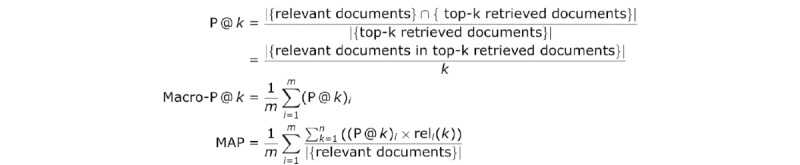
Evaluation metrics of recommendation performance.

We asked another expert to assign a recommendation score to each document in the test collection for each patient, inspired by Zeng et al [37]. The other expert reviewed the results. For the pairing of patient data *p* and educational material document *d*, the expert assigned a score in the range of 0 to 2 to indicate if *d* was appropriate to recommend to *p*, where 0 indicated no need, 1 partial need, and 2 most need. According to the inner products between the patient vector and text vectors, a ranked sequence of the test collection was returned by the system for each patient. System performance was evaluated based on the precision of top *k* retrieved documents, as shown in Figure 7, where a partial need document was counted as 0.5. Since different patients have different precisions at *k*, we used the macro precision and the overall mean average precision (MAP) to evaluate the system performance, as shown in Figure 7, where *m* corresponds to the total number of patients (*m*=50), *n* corresponds to the total number of retrieved documents (*n*=100), (P @ *k*)*_i_* corresponds to the precision at *k* for patient *i*, rel*_i_* (*k*) is an indicator function equaling 1 if the item at rank *k* is a relevant document, zero otherwise (for patient *i*).

## Results

### Overall Statistics

#### Patient Statistics

Table 2 shows a summary of the collected patient data. The patients were 50 adults with an average age of 57 years. Their characteristics were divided into 5 categories: demographics, disease history, laboratory tests, self-monitoring, and questionnaires. Among these categories, demographic data, disease history data, and laboratory test data came from the patients’ corresponding electronic health records, while questionnaire and self-monitoring data came from the patients’ daily use records of the system. For self-monitoring data, we extracted the most recent week’s records for each patient (by the end of July 2019). For questionnaire data, the 9-item Patient Health Questionnaire [51] and International Physical Activity Questionnaire [52] were used to assess the depression level and physical activity level of patients, respectively. We extracted the latest record of each patient’s questionnaire data. In recommendation generation, all the patient data were mapped to the ontology vector space with a severity level ranging from 0 to 3.

**Table 2:** Patient characteristics from the collected data (n=50).

Patient characteristics			Value
**Demographic**			
	**Sex, n (%)**		
		Female	23 (46)
		Male	27 (54)
	Age in years, mean (SD)		57 (0.57)
	**Body mass index (kg/m ^2^), n (%)**		
		Normal^a^	16 (32)
		Overweight	34 (68)
	**Pregnancy, n (%)**		
		Pregnant	0 (0)
		Nonpregnant	50 (100)
**Disease history, n (%)**			
	Hypertension		50 (100)
	Diabetes		6 (12)
	Stroke		4 (8)
	Hyperlipidemia		12 (24)
	Coronary artery disease		3 (6)
	Chronic obstructive pulmonary disease		2 (4)
	Other diseases		17 (34)
**Laboratory test, n (%)**			
	Blood glucose (normal)^b^		36 (72)
	Total cholesterol (normal)^c^		36 (72)
	Triglyceride (normal)^d^		29 (58)
	High density lipoprotein (normal)^e^		43 (86)
	Low density lipoprotein (normal)^f^		40 (80)
	Uric acid (normal)^g^		39 (78)
**Self-monitoring data, n (%)**			
	**Blood pressure**		
		Normal^h^	23 (46)
		Abnormal	27 (54)
	**Smoking and drinking**		
		Smoking	7 (14)
		Drinking	9 (18)
	**Diet**		
		Good	19 (38)
		Medium	27 (54)
		Poor	4 (8)
	**Medication**		
		Antihypertensive drugs	50 (100)
		Hypoglycemic drugs	3 (6)
		Hypolipidemic drugs	12 (24)
**Questionnaire, n (%)**			
	**9-item Patient Health Questionnaire**		
		Minimal depression	33 (66)
		Mild depression	12 (24)
		Moderate depression	3 (6)
		Moderately severe depression	2 (4)
		Severe depression	0 (0)
	**International Physical Activity Questionnaire**		
		High physical activity level	18 (36)
		Moderate physical activity level	23 (46)
		Low physical activity level	9 (18)

^a^Reference range of body mass index: 18.5-23.9 kg/m^2^ for Chinese patients.

^b^Reference range of blood glucose: 3.9-6.1 mmol/L.

^c^Reference range of total cholesterol: 2.9-5.2 mmol/L.

^d^Reference range of triglyceride: 0.56-1.70 mmol/L.

^e^Reference range of high density lipoprotein: 1.20-1.68 mmol/L.

^f^Reference range of low density lipoprotein: 2.07-3.12 mmol/L.

^g^Reference range of uric acid: 149-416 μmol/L (for men under 60), 89-357 μmol/L (for women under 60), 250-476 μmol/L (for men over 60), 190-434 μmol/L (for women over 60).

^h^Reference range of blood pressure: 90-119 mm Hg for systolic BP, 60-79 mm Hg for diastolic BP.

#### Material Statistics

**Figure 8:**
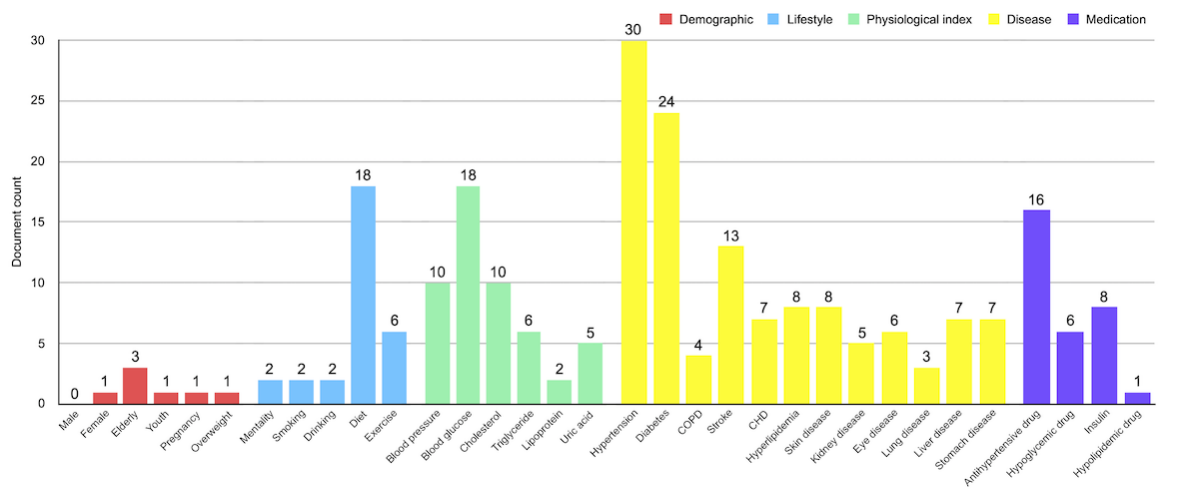
Topic distribution of the test collection.

Table 3 shows an overview of the entire corpus (88,746 documents) and the test collection (100 documents). The mean document length (word count) was 490 (SD 387) and 719 (SD 462) for the corpus and the test collection, respectively. The unique word count in the entire corpus was 270,591 with 10,707 in the test collection. Figure 8 shows the topic distribution of the test collection, in which we counted the number of documents related to each term in the ontology vector space based on the mapping method mentioned in stage 2. Among the 33 terms, hypertension, diabetes, diet, blood glucose, and antihypertensive drug were the most common topics discussed by educational materials in the test collection.

**Table 3:** Overview of the entire corpus and the test collection.

Corpus	Number	Total word count	Word count, mean (SD)	Unique word count
Entire corpus	88,746	40,797,062	490 (387)	270,591
Test collection	100	71,905	719 (462)	10,707

### System Development Results

#### Stage 1: Ontology Construction

**Figure 9:**
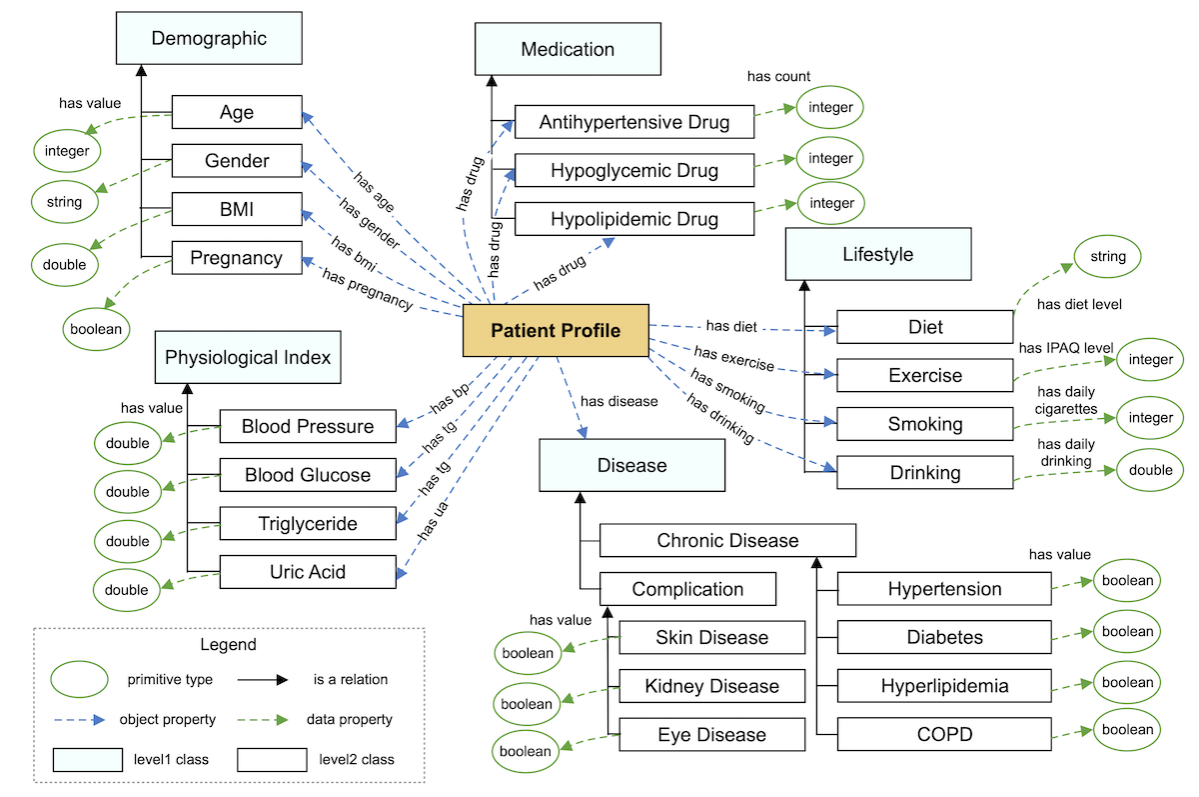
Class diagram of the Chronic Disease Patient Education Ontology’s main core.

The current version of CDPEO contained 40 classes, 31 object properties, 67 data properties, and 32 individuals (see Multimedia Appendix 4 for the detailed ontology nonzero metrics). As mentioned before, CDPEO mainly consisted of two levels: 5 terms that described patient characteristics (level 1) and the detailed elements for each term (level 2). The patient profile class was used to generate the instance of patient data, using object properties to connect to specific characteristic instances. These properties were also described as a set of universal restrictions to specify the complete semantics. All specific characteristic instances contained one or more data properties to describe the concrete value of that characteristic. For example, for blood pressure, the data property connected to two double-type values representing systolic and diastolic BP; while for smoking, the data property connected to an integer type value that describes the daily cigarette count of the patient. Figure 9 shows the class diagram of CDPEO’s main core. We have not added all the classes and properties in order to keep the figure simple. CDPEO is publicly available and can be freely downloaded from BioPortal [53].

The 33-dimensional ontology vector space was generated from the level 2 classes, in which each dimension corresponds to a term describing patient characteristics and document topics (originally in Chinese), as shown in Figure 8. A total of 80 SWRL rules were defined to implement the semantic logic of mapping patient original data to the ontology vector space. The complete SWRL list can be found in Multimedia Appendix 4 as well.

#### Stage 2: Recommendation Generation

The patient vectors were generated by the SWRL rules. In CDPEO, the 33 vector items corresponded to 33 data properties, each with a prefix of vectorItem. The general reasoning procedures of the SWRL rules are as follows: first, the rules took the patient profile instance and the connected characteristic instances (including the specific data values) as inputs and calculated the values of the vector items by using the built-in attributes to perform the logic judgment; second, the rules connected the item values to the patient profile instance via the data properties prefixed with vectorItem.

**Figure 10:**
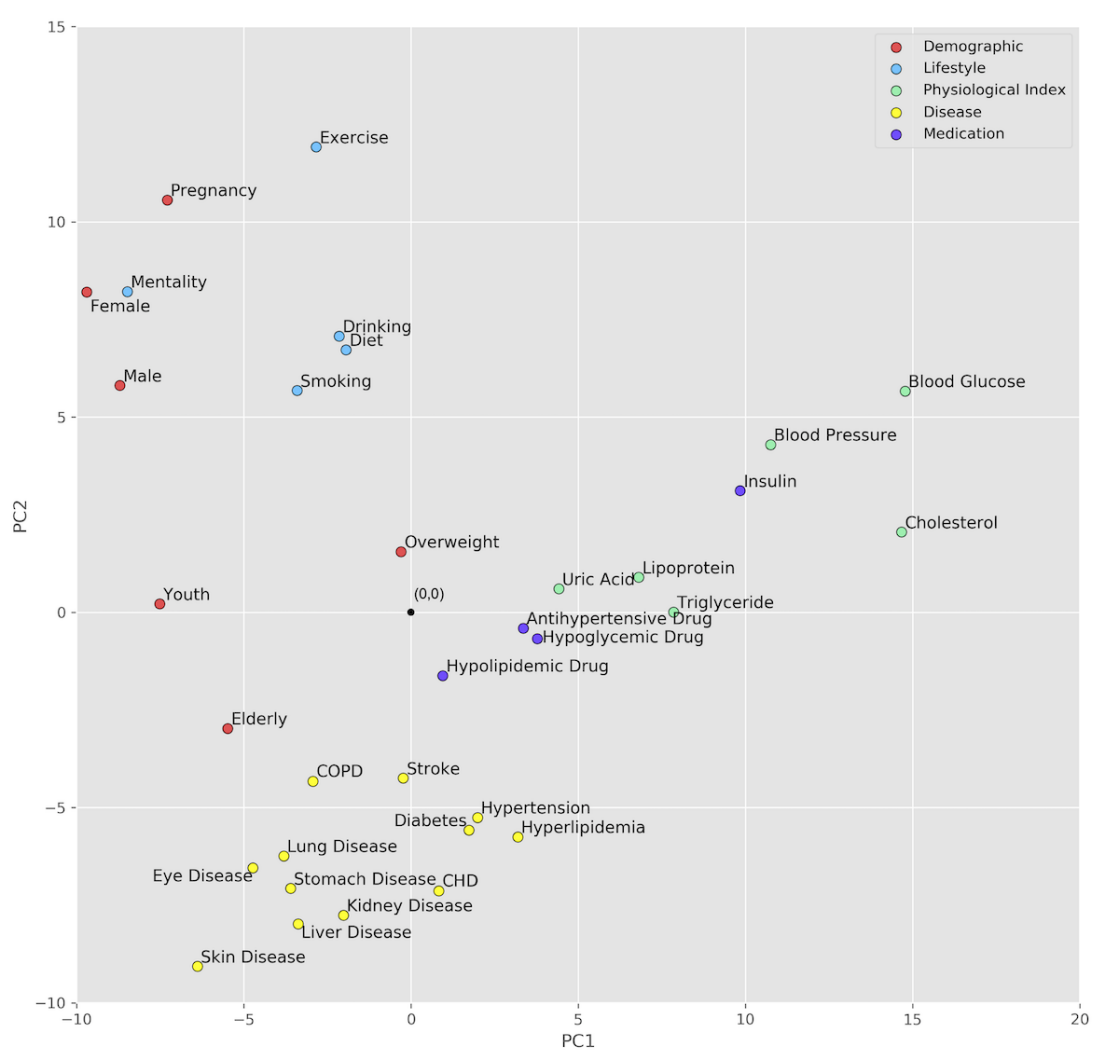
Word2Vec embedding visualization of the 33 ontology vector items.

The text vectors were generated based on the keywords of each document and the Word2Vec embeddings. The keyword extraction performance was evaluated in the next section. The pretrained embedding for each word in the corpus was a 200-dimensional vector. To intuitively evaluate the performance of the Word2Vec model, we extracted the embeddings of the 33 terms in the ontology vector space, and then visualized them in a 2-dimensional space using principal component analysis [54,55]. As shown in Figure 10, terms with similar meanings tended to have embeddings with similar directions (eg, male and female), which proves that the pretrained Word2Vec embeddings were able to capture the semantic meanings behind the words. Therefore, we used the Word2Vec embeddings to map the extracted keywords to the ontology vector space.

**Figure 11:**
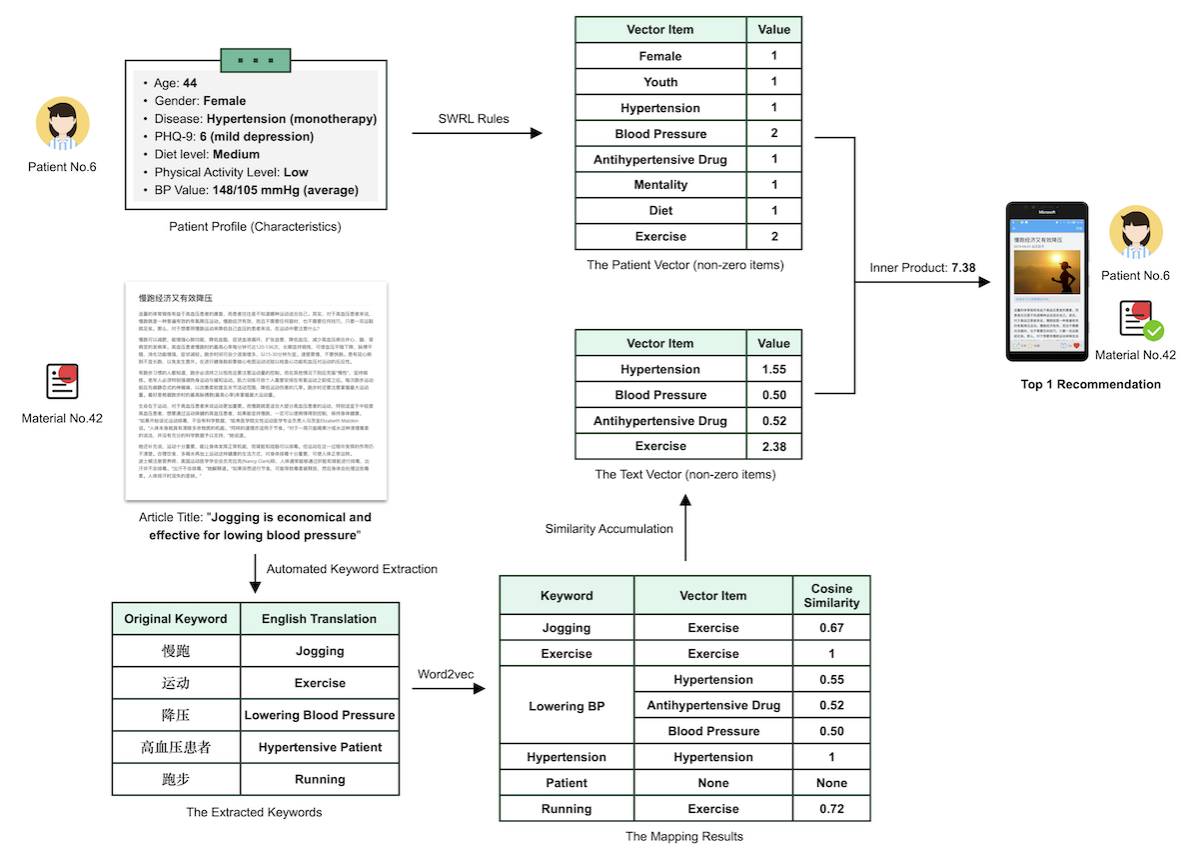
Complete scenario for the recommendation generation process.

To better understand the entire recommendation generation process, we selected one patient from the 50 and one document from the test collection to perform a simple case study. Figure 11 shows the complete scenario. The detailed description of this case study can be found in Multimedia Appendix 5.

#### Stage 3: mHealth Implementation

**Figure 12:**
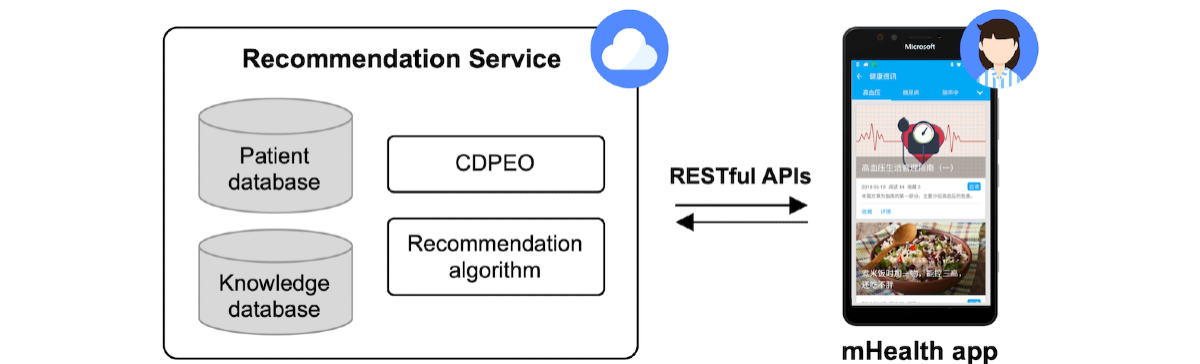
Structure of the system.

The recommender system was implemented as a part of our mHealth system [39]. Figure 12 shows the structure of the system. We designed the recommender system as a separate service, interacting with the app via RESTful APIs [56]. As mentioned before, the recommendations were first generated based on a certain threshold, and then added to a recommendation queue waiting to be pushed. In practice, the threshold is configurable, which means care providers can adjust the threshold value based on the actual effect. Considering the sparsity of the vectors, the initial threshold value was relatively small (*v*=2). The system will push the relevant documents to patients’ smartphones every day according to the recommendation queue, and update the patient and text vectors to add new relevant documents to the queue. Recommendations will be displayed in the Health Education functional module of the app.

### System Evaluation

#### Evaluation of Keyword Extraction Performance

We extracted 5 keywords for each document in the test collection automatically using 4 different algorithms. The 4 different algorithms were the original TF-IDF and TextRank methods, as well as the modified versions of them with our proposed 3 strategies. The evaluation results are shown in Table 4. Among the 4 algorithms, the improved TextRank achieved the best overall precision of 53.2% (266/500), while the improved TF-IDF achieved the worst overall precision of 26.6% (133/500).

**Table 4:** Results for automatic keyword extraction using different algorithms.

Method	Automatic extraction		Correct keywords		Precision (%)	
	Total	Mean	Total	Mean		
Improved TextRank	500	5	266	2.66		53.2
Original TextRank	500	5	151	1.51		30.2
Improved TF-IDF^a^	500	5	133	1.33		26.6
Original TF-IDF	500	5	206	2.06		41.2

^a^TF-IDF: term frequency–inverse document frequency.

#### Evaluation of Recommendation Performance

**Figure 13:**
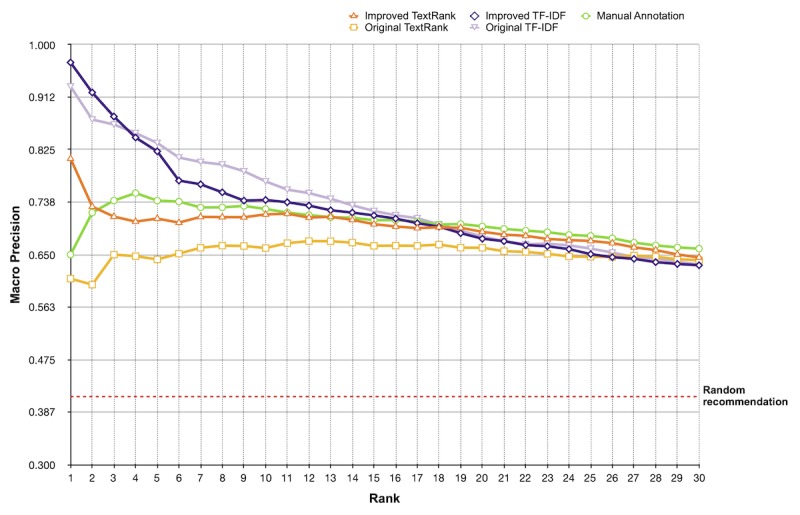
Macro precisions at rank 1 to 30 of different keyword extraction algorithms for n=50 evaluations (patients).

Based on patient data and extracted keywords for each document in the test collection, we calculated the inner products for each patient-document pair and generated the top *k* recommendations. System performance with different extracted keywords is presented in Table 5 and Figure 13. The average number of manually annotated appropriate documents for each patient was 41 out of 100, which can be considered as the macro precision for a random recommendation (the dotted red line in Figure 13).

Among the 5 methods, the improved TF-IDF achieved the highest macro precision (0.970) at the top 1 recommendation. From the curve, the TF-IDF methods (original and improved version) outperformed the TextRank methods, especially at top 1 to 10 recommendations. The manual annotated keywords had a medium performance at top 1 to 15 recommendations, compared with other methods. As the number of recommendations increases, the performances of each method tended to be closer. For the overall MAP score, manual annotation achieved the highest value (0.635), while the original TextRank achieved the lowest value (0.585). The other 3 methods obtained similar scores (mean 0.623).

**Table 5:** Performance comparison among different keyword extraction algorithms for n=50 evaluations (patients).

Method	MAP^a^	Macro Precision									
		P@1	P@2	P@3	P@4	P@5	P@10	P@15	P@20	P@25	P@30
Improved TextRank	0.622	0.810	0.730	0.713	0.705	0.710	0.717	0.701	0.688	0.673	0.645
Original TextRank	0.585	0.610	0.600	0.650	0.648	0.642	0.661	0.665	0.662	0.646	0.641
Improved TF-IDF^b^	0.620	0.970	0.920	0.880	0.845	0.822	0.741	0.715	0.677	0.651	0.632
Original TF-IDF	0.628	0.930	0.875	0.867	0.853	0.836	0.772	0.723	0.680	0.660	0.634
Manual Annotation	0.635	0.650	0.720	0.740	0.753	0.740	0.726	0.707	0.697	0.681	0.660

^a^MAP: mean average precision.

^b^TF-IDF: term frequency–inverse document frequency.

## Discussion

### Principal Findings

In this study, we investigated the use of knowledge-based RAs with a combination of NLP techniques to recommend Chinese educational materials to chronic disease patients. The constructed ontology (CDPEO) can describe patient characteristics, linking them to the topics of educational materials. The recommender system was implemented as a Web service connected with patients’ smartphones. According to the evaluation results, our system achieved a macro precision up to 0.970 for the top 1 recommendation and an overall MAP score up to 0.628.

Some interesting aspects can be found from the evaluation results. First, the improved TextRank has the potential to be the most suitable keyword extraction algorithm for our system, since it achieved the best performance in keyword extraction and obtained a relatively high score (0.622) for the overall MAP. Second, for the improved TF-IDF, there existed a performance gap between keyword extraction and recommendation. This algorithm achieved the worst performance in keyword extraction; however, it outperformed the other methods in the macro precision of the top 1 to 3 recommendations. This result may be explained by the fact that the improved TF-IDF produced output that tended toward compound words, according to the concrete extraction results. The manually extracted keywords didn’t involve many compound words, which resulted in the low precision of keyword extraction for the improved TF-IDF; however, compound words contained more information than atom words, which is advantageous for recommendation.

Third, as mentioned in the results section, the TextRank methods didn’t perform as well as the TF-IDF methods in the macro precision of recommendation. This result could be attributed to the different principles behind the two types of methods. In TF-IDF, keywords were extracted based on term frequency and inverse document frequency [44], which uses the information of the entire corpus. Further, our compound word identification strategy took the term frequency as an important filter condition, which resulted in the large amount of compound words in the keywords extracted by the improved TF-IDF method. On the other hand, the rationale of TextRank is extracting keywords based on a graph-based ranking model [45], which only uses the information of one single document. The information gap between these methods may lead to the different recommendation performances. To summarize, from our results, the performance of keyword extraction didn’t exactly correspond to the recommendation performance. Our strategies for keyword extraction have different effects on different algorithms. The recommendation performance is closely related to the rationales behind the algorithms.

### Comparison With Prior Work

To better delineate the contribution of this paper, we compared our study with prior work in two aspects. In terms of keyword extraction, several studies have explored the effect of traditional techniques combined with improvement strategies to extract keywords from Chinese documents. Li et al [57] proposed a new keyword extraction method for news documents based on TF-IDF with multistrategies. They first performed word segmentation to obtain candidate keywords of uni-, bi- and trigrams, meanwhile recognizing unknown candidate keywords based on several measures; they then calculated the features of keyword candidates to get the final keywords according to their morphological characters and context information. Wang et al [58] designed a hybrid keyword extraction method based on TF and semantic strategies. Similarly, they obtained candidate keywords based on word segmentation results and a new word-finding method, and then performed feature calculation for each candidate word and introduced several strategies to filter dependent words and remove synonyms. Zhao et al [59] applied semantic similarity computation and the frequent pattern growth algorithm to mine candidate keyword sets, and then calculated the weight of each candidate word based on frequency, part of speech, and position information.

Compared with these studies, our strategies for keyword extraction focused on patient educational materials, and extracted keywords were used as inputs for recommendation generation. The different application scenarios and objectives led to the difference in implementation details of our strategies. In weight assignment, we considered the part of speech (nouns and verbs) and the position of words (in titles) together while prior studies usually treated these items separately. In compound word identification, we recognized the compound words before the formal word segmentation and generated a user-defined dictionary to customize the word segmentation, while prior studies chose to identify such unknown words after word segmentation. In synonym elimination, we identified the synonyms by calculating the cosine similarity between character compositions of keywords, which had not been proposed in prior studies. Furthermore, we applied our strategies to two different algorithms. From the evaluation results, these strategies had a better effect on the TextRank algorithm than the TF-IDF algorithm.

In terms of the entire recommender system, several studies concerned with HRS for patient education have been described in the introduction section. Compared with these studies, our study was innovative in a few ways. First, our system was designed as a knowledge-based HRS, using ontologies to model patient characteristics, while prior studies generally adopted an IR approach [20,37] or traditional RAs [21,25,38]. Second, to the best of our knowledge, this study is the first to explore the feasibility of recommending Chinese materials using information technologies in the field of patient education. Since preprocessing procedures for Chinese documents are quite different from English documents (eg, the word segmentation), our method can provide constructive guidance to future research.

### Strengths and Limitations

Our study has several strengths. First, the vectors used for recommendation generation is adaptable to specific requirements. With simple adjustment of ontology vector space by adding or deleting terms, patient and text vectors will be automatically generated. Second, recommendations generated through our method are more interpretable than traditional methods (such as content-based methods and collaborative filtering). In traditional methods, results are generated according to users’ previous ratings, which lacks a strong explanation for the current recommendations. However, in our study, the results can be explained based on the co-occurrent nonzero items in these two vectors. A larger product of the corresponding item indicates a higher relevance of the specific characteristic (topic) between the patient and the educational item. Third, the system was implemented as a Web service of our mHealth system. Patients are able to view daily updates about personalized health information on their smartphones, which provides possibility for large-scale practical application and evaluation in the future.

A number of potential methodological weaknesses need to be acknowledged. First, the NLP techniques used in this study are mainly word-level techniques, which may not be able to capture the deep semantic meanings behind sentences or documents. The keyword extraction algorithms are word-level statistical methods and the Word2Vec model produces static word embeddings instead of contextual word embeddings. Moreover, the precision of keyword extraction remains to be improved. Second, the constructed ontology and SWRL rules remain to be further validated for their consistency, correctness, and completeness.

In addition, validity of the test collection was limited as well. Potential selection bias may exist in terms of patients and educational materials. According to the statistics, the average age of the patients was 57 years, which means the effect of our recommender system for younger patients is unknown; the mean length of the selected materials was greater than the entire corpus, which means the effect of our method on shorter text needs further investigation. Moreover, the scale of the test collection was relatively small, and manual annotation was completed by two experts separately without strict validation. The precision of 1-patient recommendation may have a great impact on the macro precision and overall MAP score.

### Future Work

In future work, we will test the effect of our method on a larger test collection. Comparison tests should be conducted to determine if our system can perform well at a larger scale. We also plan to evaluate the system for patients in a broader age distribution and involve patients in the assessment procedures. Currently, evaluation of relevance is done by case managers only, and their opinions may differ from the patients’ perceived usefulness. The opinions of patients can be used to strengthen the recommender system as well. Another direction for future work is to explore a new sentence-level or document-level approach to understand the deep semantic meanings of the materials. For example, the feasibility of applying a pretrained language model (such as bidirectional encoder representation from transformers [60] and XLNet [61]) combined with a downstream task (such as multilabel classification) would be investigated.

### Conclusions

This study has shown that a knowledge-based recommender system has the potential to accurately recommend health educational materials to chronic disease patients. Patient characteristics can be linked to document topics through the ontology. NLP techniques such as keyword extraction and pretrained word embeddings proved to be effective for processing educational materials. Furthermore, documents in Chinese have different preprocessing procedures from those in English. Our study indicates that traditional techniques combined with several strategies for specific language and domain can improve the final results to a certain extent. Further research might investigate the use of other state-of-the-art NLP techniques in HRS for better precision or explore the effect of such systems from the perspective of patients in a practical setting.

## Appendix

Multimedia Appendix 1 Detailed information about material sources.

Multimedia Appendix 2 Detailed outputs in the first three steps of the Chronic Disease Patient Education Ontology construction.

Multimedia Appendix 3 Examples of the three improvement strategies for keyword extraction.

Multimedia Appendix 4 Detailed metrics and Semantic Web Rule Language rule list of the Chronic Disease Patient Education Ontology.

Multimedia Appendix 5 Case study of the recommendation generation.

## Abbreviations

API: application programming interface
BP: blood pressure
CDPEO: Chronic Disease Patient Education Ontology
HRS: health recommender system
IR: information retrieval
MAP: mean average precision
mHealth: mobile health
NLP: natural language processing
OWL: W3C Web Ontology Language
RA: recommendation algorithm
SNOMED-CT: Systematized Nomenclature of Medicine–Clinical Terms
SWRL: Semantic Web Rule Language
TF-IDF: term frequency–inverse document frequency
