# Supplementary material for: Using Natural Language Processing Techniques to Provide Personalized Educational Materials for Chronic Disease Patients in China: Development and Assessment of a Knowledge-Based Health Recommender System
Source: JMIR Med Inform. 2020 Apr 23;8(4):e17642. doi: 10.2196/17642 (PMC7206519; doi:10.2196/17642)
Supplement: Multimedia Appendix 1 [file medinform_v8i4e17642_app1.pdf]

Detailed Information about material sources

Patient education materials used in this study came from multiple sources including websites, guidelines and books, which have been reviewed and approved by several physicians. The concrete source list are as follows:

Websites:

1. 丁香医生网（https://dxy.com/column）

Translation: Dingxiang Doctor Website (https://dxy.com/column)

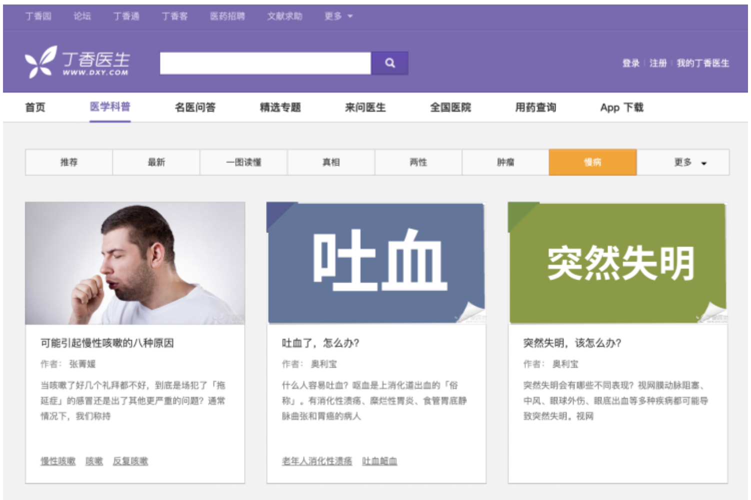

2. 39 健康网（http://www.39.net/）

Translation: 39 Health Website (http://www.39.net/)

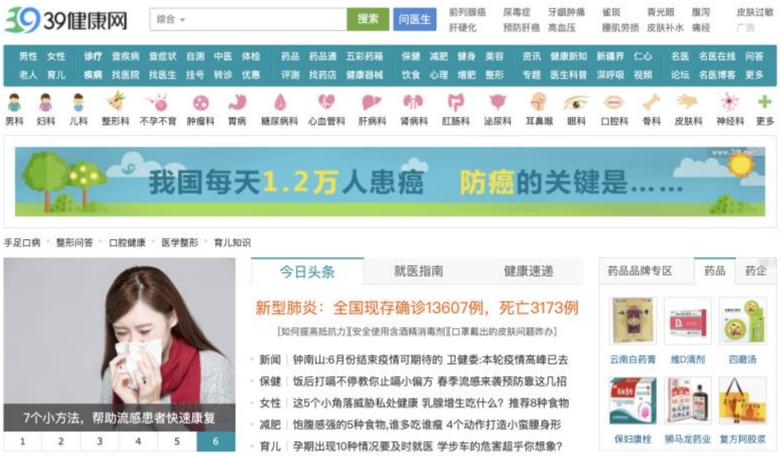

Guidelines:

1. 中国高血压患者教育指南（2013）

Translation: Chinese Guidelines of Hypertensive Patient Education (2013)

Download link: http://resource.heartonline.cn/20150515/17_x2GedVq.pdf

2. 中国糖尿病护理及教育指南（2010）
Translation: Chinese Guidelines of Care and Education in Diabetes Mellitus (2010)
Download link:
http://www.diab.net.cn/UploadFile/Ueditor/file/20160811/6360650900034000003924937.pdf

3. 中国糖尿病运动治疗指南（2014）
Translation: Chinese Guidelines of Exercise Therapy in Diabetes Mellitus (2014)
Download link:
http://www.diab.net.cn/UploadFile/Ueditor/file/20160811/6360650891598062506418654.pdf

Books:

1. 高血压日常调养专家指导全方案（2016）
Translation: Hypertension Daily Care Guidance from Experts (2016)
ISBN: 9787121275128

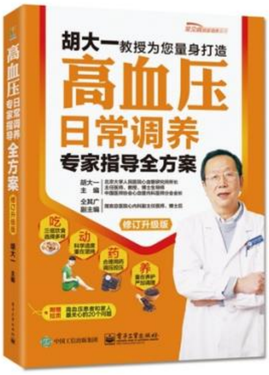

2. 糖尿病日常调养专家指导全方案（2016）
Translation: Diabetes Daily Care Guidance from Experts (2016)
ISBN: 9787121275135

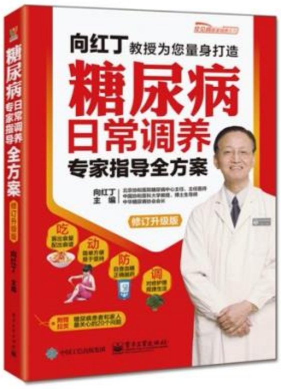

3. 一本拿下高血压（2015）

Translation: Hypertension all in one book (2015)
ISBN: 9787553701417

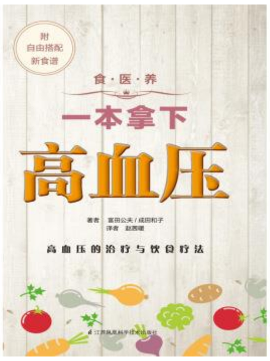

4. 一本拿下糖尿病（2015）
Translation: Diabetes all in one book (2015)
ISBN: 9787553701400

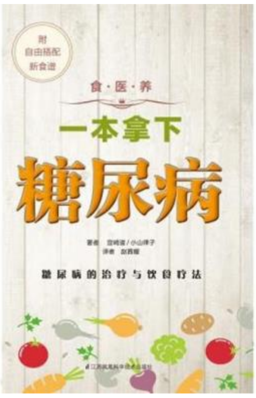

We retrieved a total of 88746 documents in Chinese from these sources. Among these documents, 511 of them were manually extracted from the guidelines or books, while the others were crawled from the websites.
