# Supplementary material for: Using Natural Language Processing Techniques to Provide Personalized Educational Materials for Chronic Disease Patients in China: Development and Assessment of a Knowledge-Based Health Recommender System
Source: JMIR Med Inform. 2020 Apr 23;8(4):e17642. doi: 10.2196/17642 (PMC7206519; doi:10.2196/17642)
Supplement: Multimedia Appendix 2 [file medinform_v8i4e17642_app2.pdf]

Detailed outputs in the first 3 steps of CDPEO construction

The first 3 steps of CDPEO construction are:
1. Determine the domain and scope of CDPEO
2. Consider reusing existing ontologies
3. Enumerate important terms in CDPEO

Detailed outputs in Step 1: List of Competency Questions:
These questions can be divided into two types: general questions and specific questions.

General questions include:
- Why build this ontology?
- What are the domains this ontology will cover?
- Who are the intended users of the ontology?
- What will this ontology be used for?
- What is the intended output of this ontology?
- What will the ontology use to make decisions?
- What resources will be considered to build the ontology?

Specific questions include:
- What are the specific patient characteristics required to recommend educational materials?
- What is the relation between patient characteristics and document topics?
- What are the main components of the intended label set?
- How do you customize a patient vector according to a patient profile?

Detailed outputs in Step 2: Searching Result of Existing Ontologies:
We searched for reusable existing ontologies on BioPortal (https://bioportal.bioontology.org/ontologies) using keywords "hypertension", "diabetes", "chronic disease," and "patient education". A total of 9 ontologies were screened.
- Hypertension Ontology (HTN)

The Hypertension Ontology is a realism-based reference ontology for semantically managing clinical data about hypertension.

- EmpowerBP (EBP)

Clinical practice guidelines, behavior change theories, and associated behavior change strategies for the management of hypertension.

- Diabetes Mellitus Diagnosis Ontology (DDO)

An ontology for diagnosis of diabetes containing the diabetes related complications, symptoms, drugs, lab tests, etc.

➢ Diabetes Mellitus Treatment Ontology (DMTO)

DMTO is an OWL 2 ontology for creating customized treatment plans for diabetic patients.

➢ HL7 FHIR & SSN ontology-based Type 1 Diabetes Mellitus Ontology (FASTO)

FHIR And SSN based Type 1 diabetes Ontology (FASTO) is an OWL 2 ontology for real time management of insulin for diabetes patients especially type 1 diabetics.

➢ BioMedBridges Diabetes Ontology (DIAB)

A diabetes ontology.

➢ Ontology of Glucose Metabolism Disorder (OGMD)

Including the disease names, phenotypes and their classifications involved in Glucose Metabolism Disorder, Diabetes.

➢ Chronic Kidney Disease Ontology (CKDO)

The Chronic Kidney Disease Ontology was developed to assist routine data studies and case identification of CKD in primary care.

➢ COPD Ontology (COPDO)

The COPD Ontology is a biomedical ontology used for modelling concepts associated to chronic obstructive pulmonary disease in routine clinical databases.

Detailed outputs in Step 3: List of Collected Terms:

➢ 患者 (Patients)	➢ 怀孕 (Pregnancy)
➢ 疾病 (Disease)	➢ 男性 (Male)
➢ 慢性疾病 (Chronic Disease)	➢ 女性 (Female)
➢ 高血压 (Hypertension)	➢ 老年 (Elderly)
➢ 糖尿病 (Diabetes)	➢ 青年 (Youth)
➢ 慢阻肺 (COPD)	➢ 超重 (Overweight)
➢ 冠心病 (Coronary Heart Disease)	➢ 生活方式 (Disease)
➢ 高血脂 (Hyperlipidemia)	➢ 吸烟 (Smoking)
➢ 脑卒中 (Stroke)	➢ 饮酒 (Drinking)
➢ 肾病 (Kidney Disease)	➢ 饮食 (Diet)
➢ 皮肤病 (Skin Disease)	➢ 运动 (Exercise)
➢ 眼病 (Eye Disease)	➢ 心理 (Mentality)
➢ 肺病 (Lung Disease)	➢ 生理参数 (Physiological Index)
➢ 肝病 (Liver Disease)	➢ 血压 (Blood Pressure)
➢ 胃病 (Stomach Disease)	➢ 血糖 (Blood Glucose)
➢ 人口学资料 (Demographic)	➢ 胆固醇 (Cholesterol)
➢ 年龄 (Age)	➢ 甘油三酯 (Triglyceride)
➢ 性别 (Sex)	➢ 高密度脂蛋白 (HDL)
➢ BMI	➢ 低密度脂蛋白 (LDL)

➢ 问卷调查 (Questionnaire)	➢ 尿酸 (Uric Acid)
➢ 实验室测试 (Lab test)	➢ 收缩压 (Systolic BP)
➢ 自我监测 (Self-monitoring)	➢ 舒张压 (Diastolic BP)
➢ 用药 (Medication)	➢ 每日吸烟 (Daily Smoking)
➢ 降压药 (Antihypertensive drug)	➢ 每日饮酒 (Daily Drinking)
➢ 降糖药 (Hypoglycemic drug)	➢ 并发症 (Complication)
➢ 降脂药 (Hypolipidemic drug)	➢ IPAQ
➢ 胰岛素 (Insulin)	➢ PHQ-9
