# Supplementary material for: Using Natural Language Processing Techniques to Provide Personalized Educational Materials for Chronic Disease Patients in China: Development and Assessment of a Knowledge-Based Health Recommender System
Source: JMIR Med Inform. 2020 Apr 23;8(4):e17642. doi: 10.2196/17642 (PMC7206519; doi:10.2196/17642)
Supplement: Multimedia Appendix 3 [file medinform_v8i4e17642_app3.pdf]

Examples of the 3 improvement strategies for keyword extraction

The 3 strategies introduced for keyword extraction are:
1. Weight assignment
2. Compound word identification
3. Synonym elimination

We will illustrate the effect of these 3 strategies based on a patient education material entitled “一杯黄芪水的功效” (The Effect of A Cup of Astragalus Water).

Example of Weight Assignment:
In weight assignment, we set an additional weight value for the title words (a weight of 3), the nouns (a weight of 1.2) and the verbs (a weight of 0.8) in the materials. In the sample material, the title words include: “一杯” (A cup), “黄芪” (Astragalus), “水” (Water) and “功效” (Effect), which will be set an additional weight value of 3. We perform the keyword extraction using the TextRank algorithm combined with this strategy. According to the extraction result of 5 keywords, the title term “黄芪” (Astragalus), “水” (Water) and “功效” (Effect) are selected as the keywords.

Example of Compound Word Identification:
In compound word identification, we set several filter conditions to identify compound words in patient education materials and generate a user-defined dictionary to customize the word segmentation. We perform the keyword extraction using the TextRank algorithm combined with the above 2 strategies. In the sample material, two compound words are identified as the new keywords: “黄芪泡水” (Astragalus bubble water) and “黄芪水” (Astragalus water).

Example of Synonym Elimination:
In synonym elimination, we remove shorter keywords with similar Chinese characters based on the cosine similarity between their character compositions. Concretely, if the cosine similarity is greater than a threshold, then the 2 keywords will be considered as a synonym pair. The threshold here is set as 0.7. In the sample material, the one-hot vectors for the two compound keywords and their cosine similarity are as follows:

$$\overrightarrow{A} = \{\text{黄芪泡水：黄 1、芪 1、泡 1、水 1}\} = [1,1,1,1]$$

$$\overrightarrow{B} = \{\text{黄芪水：黄 1、芪 1、泡 0、水 1}\} = [1,1,0,1]$$

$$\cos(\theta) = \frac{\overrightarrow{A} \cdot \overrightarrow{B}}{||\overrightarrow{A}||||\overrightarrow{B}||}$$

The cosine similarity is 0.866, which is greater than 0.7. Therefore, we keep the term “黄芪泡水” (Astragalus bubble water) as the keyword and remove the shorter term “黄芪水” (Astragalus water).
