# Supplementary material for: Using Natural Language Processing Techniques to Provide Personalized Educational Materials for Chronic Disease Patients in China: Development and Assessment of a Knowledge-Based Health Recommender System
Source: JMIR Med Inform. 2020 Apr 23;8(4):e17642. doi: 10.2196/17642 (PMC7206519; doi:10.2196/17642)
Supplement: Multimedia Appendix 4 [file medinform_v8i4e17642_app4.pdf]

Table S1. CDPEO ontology metrics

Metric		Count
**Overall Metrics**		
	Classes	40
	Axioms	799
	Logical Axioms	630
	Declaration Axioms	169
	Object Properties	31
	Data Properties	67
	Individuals	32
	SWRL Rules	80
**Class Axioms**		
	SubClassOf Axioms	64
	DisjointClasses Axioms	7
**Object Property Axioms**		
	SubObjectPropertyOf Axioms	30
	FunctionalObjectProperty Axioms	30
	ObjectPropertyDomain Axioms	30
	ObjectPropertyRange Axioms	30
**Data Property Axioms**		
	SubDataPropertyOf Axioms	66
	FunctionalDataProperty Axioms	66
	DataPropertyDomain Axioms	66
	DataPropertyRange Axioms	66
**Individual Axioms**		
	ClassAssertion Axioms	32
	ObjectPropertyAssertion Axioms	30
	DataPropertyAssertion Axioms	33

Table S2. All SWRL Rules in CDPEO

Category	Rule Number	SWRL Rule	Corresponding Vector Item
Demographics	**A01**	PatientProfile(?p) ^ hasGender(?p, ?g) ^ hasGenderValue(?g, "F") -> vectorItemFemale(?p, 1) ^ vectorItemMale(?p, 0)	Female
	**A02**	PatientProfile(?p) ^ hasGender(?p, ?g) ^ hasGenderValue(?g, "M") -> vectorItemFemale(?p, 0) ^ vectorItemMale(?p, 1)	Male
	**A03**	PatientProfile(?p) ^ hasAge(?p, ?a) ^ hasAgeValue(?a, ?v) ^ swrlb:greaterThanOrEqual(?v, 60) -> vectorItemYouth(?p, 0) ^ vectorItemOldAge(?p, 1)	Elderly
	**A04**	PatientProfile(?p) ^ hasAge(?p, ?a) ^ hasAgeValue(?a, ?v) ^ swrlb:lessThanOrEqual(?v, 60) -> vectorItemYouth(?p, 1) ^ vectorItemOldAge(?p, 0)	Youth
	**A05**	PatientProfile(?p) ^ hasPregnancy(?p, ?pr) ^ hasPregnancyValue(?pr, true) -> vectorItemPregnant(?p, 1)	Pregnancy
	**A06**	PatientProfile(?p) ^ hasPregnancy(?p, ?pr) ^ hasPregnancyValue(?pr, false) -> vectorItemPregnant(?p, 0)	Pregnancy
	**A07**	PatientProfile(?p) ^ hasBMI(?p, ?b) ^ hasBMIValue(?b, ?v) ^ swrlb:greaterThan(?v, 24) -> vectorItemOverWeight(?p, 1)	Overweight
	**A08**	PatientProfile(?p) ^ hasBMI(?p, ?b) ^ hasBMIValue(?b, ?v) ^ swrlb:lessThanOrEqual(?v, 24) -> vectorItemOverWeight(?p, 0)	Overweight
Disease	**B01**	PatientProfile(?p) ^ hasHypertension(?p, ?h) ^ hasHypertensionValue(?h, true) -> vectorItemHypertension(?p, 1)	Hypertension
	**B02**	PatientProfile(?p) ^ hasHypertension(?p, ?h) ^ hasHypertensionValue(?h, false) ->	Hypertension

		vectorItemHypertension(?p, 0)	
	**B03**	PatientProfile(?p) ^ hasCOPD(?p, ?c) ^ hasCOPDValue(?c, false) -> vectorItemChronicObstructivePulmonaryDisease(?p, 0)	COPD
	**B04**	PatientProfile(?p) ^ hasCOPD(?p, ?c) ^ hasCOPDValue(?c, true) -> vectorItemChronicObstructivePulmonaryDisease(?p, 1)	COPD
	**B05**	PatientProfile(?p) ^ hasCHD(?p, ?c) ^ hasCHDValue(?c, true) -> vectorItemCoronaryHeartDisease(?p, 1)	CHD
	**B06**	PatientProfile(?p) ^ hasCHD(?p, ?c) ^ hasCHDValue(?c, false) -> vectorItemCoronaryHeartDisease(?p, 0)	CHD
	**B07**	PatientProfile(?p) ^ hasDiabetes(?p, ?d) ^ hasDiabetesValue(?d, false) -> vectorItemDiabetes(?p, 0)	Diabetes
	**B08**	PatientProfile(?p) ^ hasDiabetes(?p, ?d) ^ hasDiabetesValue(?d, true) -> vectorItemDiabetes(?p, 1)	Diabetes
	**B09**	PatientProfile(?p) ^ hasHyperlipidemia(?p, ?h) ^ hasHyperlipidemiaValue(?h, true) -> vectorItemHyperlipidemia(?p, 1)	Hyperlipidemia
	**B10**	PatientProfile(?p) ^ hasHyperlipidemia(?p, ?h) ^ hasHyperlipidemiaValue(?h, false) -> vectorItemHyperlipidemia(?p, 0)	Hyperlipidemia
	**B11**	PatientProfile(?p) ^ hasStroke(?p, ?s) ^ hasStrokeValue(?h, false) -> vectorItemStroke(?p, 0)	Stroke
	**B12**	PatientProfile(?p) ^ hasStroke(?p, ?s) ^ hasStrokeValue(?h, true) -> vectorItemStroke(?p, 1)	Stroke
	**B13**	PatientProfile(?p) ^ hasEyeDisease(?p, ?d) ^ hasEyeDiseaseValue(?d, false) -> vectorItemEyeDisease(?p, 0)	Eye Disease
	**B14**	PatientProfile(?p) ^ hasEyeDisease(?p, ?d) ^ hasEyeDiseaseValue(?d, true) -> vectorItemEyeDisease(?p, 1)	Eye Disease
	**B15**	PatientProfile(?p) ^ hasKidneyDisease(?p, ?d) ^ hasKidneyDiseaseValue(?d, true) ->	Kidney Disease

		vectorItemKidneyDisease(?p, 1)	
	**B16**	PatientProfile(?p) ^ hasKidneyDisease(?p, ?d) ^ hasKidneyDiseaseValue(?d, false) -> vectorItemKidneyDisease(?p, 0)	Kidney Disease
	**B17**	PatientProfile(?p) ^ hasSkinDisease(?p, ?d) ^ hasSkinDiseaseValue(?d, false) -> vectorItemSkinDisease(?p, 0)	Skin Disease
	**B18**	PatientProfile(?p) ^ hasSkinDisease(?p, ?d) ^ hasSkinDiseaseValue(?d, true) -> vectorItemSkinDisease(?p, 1)	Skin Disease
	**B19**	PatientProfile(?p) ^ hasLungDisease(?p, ?d) ^ hasLungDiseaseValue(?d, true) -> vectorItemLungDisease(?p, 1)	Lung Disease
	**B20**	PatientProfile(?p) ^ hasLungDisease(?p, ?d) ^ hasLungDiseaseValue(?d, false) -> vectorItemLungDisease(?p, 0)	Lung Disease
	**B21**	PatientProfile(?p) ^ hasLiverDisease(?p, ?d) ^ hasLiverDiseaseValue(?d, false) -> vectorItemLiverDisease(?p, 0)	Liver Disease
	**B22**	PatientProfile(?p) ^ hasLiverDisease(?p, ?d) ^ hasLiverDiseaseValue(?d, true) -> vectorItemLiverDisease(?p, 1)	Liver Disease
	**B23**	PatientProfile(?p) ^ hasStomachDisease(?p, ?d) ^ hasStomachDiseaseValue(?d, true) -> vectorItemStomachDisease(?p, 1)	Stomach Disease
	**B24**	PatientProfile(?p) ^ hasStomachDisease(?p, ?d) ^ hasStomachDiseaseValue(?d, false) -> vectorItemStomachDisease(?p, 0)	Stomach Disease
**Lifestyle**	**C01**	PatientProfile(?p) ^ hasMentality(?p, ?m) ^ hasPHQ9Value(?m, ?v) ^ swrlb:lessThanOrEqual(?v, 4) -> vectorItemDepression(?p, 0)	Mentality
	**C02**	PatientProfile(?p) ^ hasMentality(?p, ?m) ^ hasPHQ9Value(?m, ?v) ^ swrlb:lessThanOrEqual(?v, 9) ^	Mentality

		swrlb:greaterThanOrEqual(?v, 5) -> vectorItemDepression(?p, 1)	
	**C03**	PatientProfile(?p) ^ hasMentality(?p, ?m) ^ hasPHQ9Value(?m, ?v) ^ swrlb:lessThanOrEqual(?v, 14) ^ swrlb:greaterThanOrEqual(?v, 10) -> vectorItemDepression(?p, 2)	Mentality
	**C04**	PatientProfile(?p) ^ hasMentality(?p, ?m) ^ hasPHQ9Value(?m, ?v) ^ swrlb:lessThanOrEqual(?v, 19) ^ swrlb:greaterThanOrEqual(?v, 15) -> vectorItemDepression(?p, 3)	Mentality
	**C05**	PatientProfile(?p) ^ hasMentality(?p, ?m) ^ hasPHQ9Value(?m, ?v) ^ swrlb:lessThanOrEqual(?v, 27) ^ swrlb:greaterThanOrEqual(?v, 20) -> vectorItemDepression(?p, 4)	Mentality
	**C06**	PatientProfile(?p) ^ hasSmoking(?p, ?s) ^ hasDailyCigarettes(?s, ?d) ^ swrlb:lessThanOrEqual(?d, 10) ^ swrlb:greaterThan(?d, 0) -> vectorItemSmoking(?p, 1)	Smoking
	**C07**	PatientProfile(?p) ^ hasSmoking(?p, ?s) ^ hasDailyCigarettes(?s, ?d) ^ swrlb:lessThanOrEqual(?d, 20) ^ swrlb:greaterThanOrEqual(?d, 11) -> vectorItemSmoking(?p, 2)	Smoking
	**C08**	PatientProfile(?p) ^ hasSmoking(?p, ?s) ^ hasDailyCigarettes(?s, ?d) ^ swrlb:greaterThanOrEqual(?d, 21) -> vectorItemSmoking(?p, 3)	Smoking
	**C09**	PatientProfile(?p) ^ hasSmoking(?p, ?s) ^ swrlb:equal(?d, 0) ^ hasDailyCigarettes(?s, ?d) -> vectorItemSmoking(?p, 0)	Smoking
	**C10**	PatientProfile(?p) ^ hasDrinking(?p, ?d) ^ hasDailyDrinking(?s, ?da) ^ swrlb:equal(?da, 0) -> vectorItemDrinking(?p, 0)	Drinking
	**C11**	PatientProfile(?p) ^ hasDrinking(?p, ?d) ^ hasDailyDrinking(?s, ?da) ^ swrlb:greaterThan(?da, 0) ^ swrlb:lessThanOrEqual(?da, 1) -> vectorItemDrinking(?p, 1)	Drinking

	**C12**	PatientProfile(?p) ^ hasDrinking(?p, ?d) ^ hasDailyDrinking(?s, ?da) ^ swrlb:lessThan(?da, 3) ^ swrlb:greaterThan(?da, 1) -> vectorItemDrinking(?p, 2)	Drinking
	**C13**	PatientProfile(?p) ^ hasDrinking(?p, ?d) ^ hasDailyDrinking(?s, ?da) ^ swrlb:greaterThanOrEqual(?da, 3) -> vectorItemDrinking(?p, 3)	Drinking
	**C14**	PatientProfile(?p) ^ hasDiet(?p, ?d) ^ hasDietLevel(?d, "good") -> vectorItemDiet(?p, 0)	Diet
	**C15**	PatientProfile(?p) ^ hasDiet(?p, ?d) ^ hasDietLevel(?d, "medium") -> vectorItemDiet(?p, 1)	Diet
	**C16**	PatientProfile(?p) ^ hasDiet(?p, ?d) ^ hasDietLevel(?d, "poor") -> vectorItemDiet(?p, 2)	Diet
	**C17**	PatientProfile(?p) ^ hasExercise(?p, ?e) ^ hasIPAQLevel(?e, "high") -> vectorItemExercise(?p, 0)	Exercise
	**C18**	PatientProfile(?p) ^ hasExercise(?p, ?e) ^ hasIPAQLevel(?e, "moderate") -> vectorItemExercise(?p, 1)	Exercise
	**C19**	PatientProfile(?p) ^ hasExercise(?p, ?e) ^ hasIPAQLevel(?e, "low") -> vectorItemExercise(?p, 2)	Exercise
**Physiological Index**	**D01**	PatientProfile(?p) ^ hasBP(?p, ?b) ^ hasSBPValue(?b, ?sv) ^ swrlb:greaterThanOrEqual(?sv, 180) -> vectorItemBloodPressure(?p, 3)	Blood Pressure
	**D02**	PatientProfile(?p) ^ hasBP(?p, ?b) ^ hasDBPValue(?b, ?dv) ^ swrlb:greaterThanOrEqual(?dv, 110) -> vectorItemBloodPressure(?p, 3)	Blood Pressure
	**D03**	PatientProfile(?p) ^ hasBP(?p, ?b) ^ hasDBPValue(?b, ?dv) ^ swrlb:greaterThanOrEqual(?dv, 100) ^ swrlb:lessThan(?dv, 110) -> vectorItemBloodPressure(?p, 2)	Blood Pressure
	**D04**	PatientProfile(?p) ^ hasBP(?p, ?b) ^ hasSBPValue(?b, ?sv) ^ swrlb:greaterThanOrEqual(?sv, 160) ^ swrlb:lessThan(?sv, 180) ->	Blood Pressure

		vectorItemBloodPressure(?p, 2)	
	**D05**	PatientProfile(?p) ^ hasBP(?p, ?b) ^ hasDBPValue(?b, ?dv) ^ swrlb:greaterThanOrEqual(?dv, 100) ^ swrlb:lessThan(?dv, 110) -> vectorItemBloodPressure(?p, 2)	Blood Pressure
	**D06**	PatientProfile(?p) ^ hasBP(?p, ?b) ^ hasDBPValue(?b, ?dv) ^ swrlb:greaterThanOrEqual(?dv, 90) ^ swrlb:lessThan(?dv, 100) -> vectorItemBloodPressure(?p, 1)	Blood Pressure
	**D07**	PatientProfile(?p) ^ hasBP(?p, ?b) ^ hasDBPValue(?b, ?dv) ^ swrlb:lessThan(?dv, 90) ^ hasSBPValue(?b, ?sv) ^ swrlb:lessThan(?sv, 140) -> vectorItemBloodPressure(?p, 0)	Blood Pressure
	**D08**	PatientProfile(?p) ^ hasBG(?p, ?b) ^ hasBGValue(?b, ?v) ^ swrlb:lessThan(?v, 6.1) -> vectorItemBloodGlucose(?p, 0)	Blood Glucose
	**D09**	PatientProfile(?p) ^ hasBG(?p, ?b) ^ hasBGValue(?b, ?v) ^ swrlb:lessThan(?v, 7) ^ swrlb:greaterThanOrEqual(?v, 6.1) -> vectorItemBloodGlucose(?p, 1)	Blood Glucose
	**D10**	PatientProfile(?p) ^ hasBG(?p, ?b) ^ hasBGValue(?b, ?v) ^ swrlb:greaterThanOrEqual(?v, 7) -> vectorItemBloodGlucose(?p, 2)	Blood Glucose
	**D11**	PatientProfile(?p) ^ hasLipoprotein(?p, ?l) ^ hasHDLValue(?l, ?h) ^ swrlb:lessThan(?h, 1.2) -> vectorItemLipoprotein(?p, 1)	Lipprotein
	**D12**	PatientProfile(?p) ^ hasLipoprotein(?p, ?l) ^ hasLDLValue(?l, ?lv) ^ swrlb:greaterThan(?lv, 3.12) -> vectorItemLipoprotein(?p, 1)	Lipprotein
	**D13**	PatientProfile(?p) ^ hasLipoprotein(?p, ?l) ^ hasLDLValue(?l, ?lv) ^ swrlb:lessThanOrEqual(?lv, 3.12) ^ hasHDLValue(?l, ?hv) ^ swrlb:greaterThanOrEqual(?hv, 1.2) -> vectorItemLipoprotein(?p, 0)	Lipprotein
	**D14**	PatientProfile(?p) ^ hasTC(?p, ?t) ^ hasTCValue(?t, ?v) ^	Total Cholesterol

		swrlb:lessThanOrEqual(?v, 5.2) -> vectorItemTotalCholesterol(?p, 0)	
	**D15**	PatientProfile(?p) ^ hasTC(?p, ?t) ^ hasTCValue(?t, ?v) ^ swrlb:greaterThan(?v, 5.2) -> vectorItemTotalCholesterol(?p, 1)	Total Cholesterol
	**D16**	PatientProfile(?p) ^ hasTG(?p, ?t) ^ hasTGValue(?t, ?v) ^ swrlb:greaterThan(?v, 1.7) -> vectorItemTriglyceride(?p, 1)	Triglyceride
	**D17**	PatientProfile(?p) ^ hasTG(?p, ?t) ^ hasTGValue(?t, ?v) ^ swrlb:lessThanOrEqual(?v, 1.7) -> vectorItemTriglyceride(?p, 0)	Triglyceride
	**D18**	PatientProfile(?p) ^ hasUA(?p, ?u) ^ hasUAValue(?u, ?v) ^ swrlb:lessThanOrEqual(?v, 416) ^ hasAge(?p, ?a) ^ hasAgeValue(?a, ?av) ^ swrlb:lessThanOrEqual(?av, 60) ^ hasGender(?p, ?g) ^ hasGenderValue(?g, "M") -> vectorItemUricAcid(?p, 0)	Uric Aid
	**D19**	PatientProfile(?p) ^ hasUA(?p, ?u) ^ hasUAValue(?u, ?v) ^ swrlb:lessThanOrEqual(?v, 357) ^ hasAge(?p, ?a) ^ hasAgeValue(?a, ?av) ^ swrlb:lessThanOrEqual(?av, 60) ^ hasGender(?p, ?g) ^ hasGenderValue(?g, "F") -> vectorItemUricAcid(?p, 0)	Uric Aid
	**D20**	PatientProfile(?p) ^ hasUA(?p, ?u) ^ hasUAValue(?u, ?v) ^ swrlb:greaterThan(?v, 416) ^ hasAge(?p, ?a) ^ hasAgeValue(?a, ?av) ^ swrlb:lessThanOrEqual(?av, 60) ^ hasGender(?p, ?g) ^ hasGenderValue(?g, "M") -> vectorItemUricAcid(?p, 1)	Uric Aid
	**D21**	PatientProfile(?p) ^ hasUA(?p, ?u) ^ hasUAValue(?u, ?v) ^ swrlb:greaterThan(?v, 357) ^ hasAge(?p, ?a) ^ hasAgeValue(?a, ?av) ^ swrlb:lessThanOrEqual(?av, 60) ^ hasGender(?p, ?g) ^ hasGenderValue(?g, "F") -> vectorItemUricAcid(?p, 1)	Uric Aid
	**D22**	PatientProfile(?p) ^ hasUA(?p, ?u) ^ hasUAValue(?u, ?v) ^	Uric Aid

		swrlb:lessThanOrEqual(?v, 434) ^ hasAge(?p, ?a) ^ hasAgeValue(?a, ?av) ^ swrlb:greaterThan(?av, 60) ^ hasGender(?p, ?g) ^ hasGenderValue(?g, "F") -> vectorItemUricAcid(?p, 0)	
	**D23**	PatientProfile(?p) ^ hasUA(?p, ?u) ^ hasUAValue(?u, ?v) ^ swrlb:lessThanOrEqual(?v, 476) ^ hasAge(?p, ?a) ^ hasAgeValue(?a, ?av) ^ swrlb:greaterThan(?av, 60) ^ hasGender(?p, ?g) ^ hasGenderValue(?g, "M") -> vectorItemUricAcid(?p, 0)	Uric Aid
	**D24**	PatientProfile(?p) ^ hasUA(?p, ?u) ^ hasUAValue(?u, ?v) ^ swrlb:greaterThan(?v, 476) ^ hasAge(?p, ?a) ^ hasAgeValue(?a, ?av) ^ swrlb:greaterThan(?av, 60) ^ hasGender(?p, ?g) ^ hasGenderValue(?g, "M") -> vectorItemUricAcid(?p, 1)	Uric Aid
	**E01**	PatientProfile(?p) ^ hasAntiHypertensiveDrug(?p, ?d) ^ hasAntiHypertensiveDrugValue(?d, ?v) -> vectorItemAntiHypertensiveDrug(?p, ?v)	Anti-hypertensive Drug
	**E02**	PatientProfile(?p) ^ hasHypoglycemicDrug(?p, ?d) ^ hasHypoglycemicDrugValue(?d, ?v) -> vectorItemHypoglycemicDrug(?p, ?v)	Hypoglycemic Drug
**Medication**	**E03**	PatientProfile(?p) ^ hasHypolipidemicDrug(?p, ?d) ^ hasHypolipidemicDrugValue(?d, ?v) -> vectorItemHypolipidemicDrug(?p, ?v)	Hypolipidemic Drug
	**E04**	PatientProfile(?p) ^ hasHypoglycemicDrug(?p, ?d) ^ hasInsulinValue(?d, true) -> vectorItemInsulin(?p, 1)	Insulin
	**E05**	PatientProfile(?p) ^ hasHypoglycemicDrug(?p, ?d) ^ hasInsulinValue(?d, false) -> vectorItemInsulin(?p, 0)	Insulin
