# Supplementary material for: Using Natural Language Processing Techniques to Provide Personalized Educational Materials for Chronic Disease Patients in China: Development and Assessment of a Knowledge-Based Health Recommender System
Source: JMIR Med Inform. 2020 Apr 23;8(4):e17642. doi: 10.2196/17642 (PMC7206519; doi:10.2196/17642)
Supplement: Multimedia Appendix 5 [file medinform_v8i4e17642_app5.pdf]

A case study of recommendation generation

To better understand the entire recommendation generation process, we selected one patient and one material to perform a simple case study. Figure 1 shows the complete scenario for the recommendation generation process. This scenario starts and ends at the patient. First, the ontology receives the patient data and generates the patient vector through the SWRL rules. In this case study, the patient is a female hypertensive patient aged 44 years with a PHQ-9 score of 6 (mild depression), a medium diet level and a low physical activity level. Currently the patient is using monotherapy, with an average blood pressure of 148/105 mmHg in the latest week. Based on the original patient data, the SWRL rules will infer each item value of the patient vector. For example, the BP value will fire the Rule D05, setting the value of the item “blood pressure” to 2. Table 1 shows the fired SWRL Rules in this case study. The non-zero items of the patient vector are “female” (value: 1), “youth” (value: 1), “hypertension” (value: 1), “blood pressure” (value: 2), “anti-hypertensive drug” (value: 1), “mentality” (value: 1), “diet” (value: 1) and “exercise” (value: 2).

Figure S1. Complete scenario for the recommendation generation process

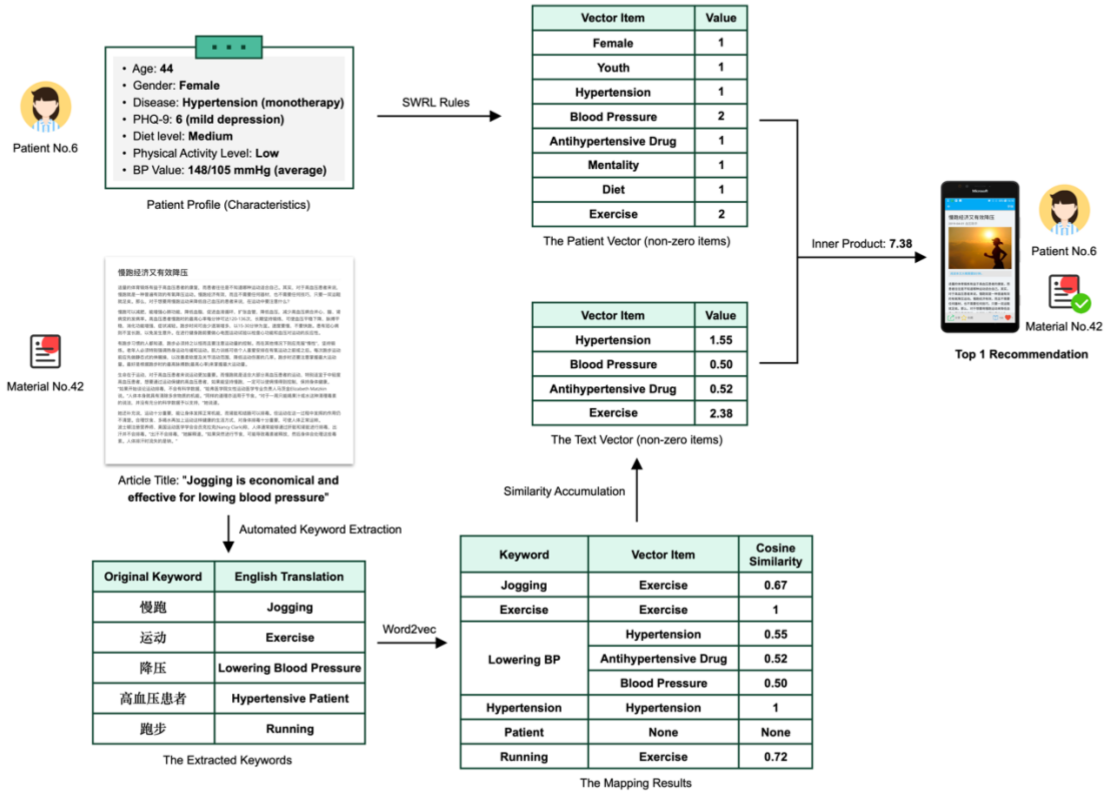

Table S3. Fired SWRL Rules in the case study

**Category**	**Original Data**	**Corresponding Vector Item**	**Fired SWRL Rule**	**Inferred Item Value**
Demographics	Female	Female	**A01**: PatientProfile(?p) ^ hasGender(?p, ?g) ^ hasGenderValue(?g, "F") -> vectorItemFemale(?p, 1) ^ vectorItemMale(?p, 0)	**1**
	Age: 44	Youth	**A04:** PatientProfile(?p) ^ hasAge(?p, ?a) ^ hasAgeValue(?a, ?v) ^ swrlb:lessThanOrEqual(?v, 60) -> vectorItemYouth(?p, 1) ^ vectorItemOldAge(?p, 0)	**1**
Disease	Hypertension	Hypertension	**B01:** PatientProfile(?p) ^ hasHypertension(?p, ?h) ^ hasHypertensionValue(?h, true) -> vectorItemHypertension(?p, 1)	**1**
Physiological Index	Average BP: 148/105 mmHg	Blood Pressure	**D05**: PatientProfile(?p) ^ hasBP(?p, ?b) ^ hasDBPValue(?b, ?dv) ^ swrlb:greaterThanOrEqual(?dv, 100) ^ swrlb:lessThan(?dv, 110) -> vectorItemBloodPressure(?p, 2)	**2**
Medication	Monotherapy	Anti-hypertensive Drug	**E01:** PatientProfile(?p) ^ hasAntiHypertensiveDrug(?p, ?d) ^ hasAntiHypertensiveDrugValue(?d, ?v) -> vectorItemAntiHypertensiveDrug(?p, ?v)	**1**
Lifestyle	PHQ-9 score: 6	Mentality	**C02:** PatientProfile(?p) ^ hasMentality(?p, ?m) ^ hasPHQ9Value(?m, ?v) ^ swrlb:lessThanOrEqual(?v, 9) ^ swrlb:greaterThanOrEqual(?v, 5) -> vectorItemDepression(?p, 1)	**1**
	Diet Level: medium	Diet	**C15:** PatientProfile(?p) ^ hasDiet(?p, ?d) ^ hasDietLevel(?d, "medium") -> vectorItemDiet(?p, 1)	**1**
	Physical Activity Level: low	Exercise	**C19:** PatientProfile(?p) ^ hasExercise(?p, ?e) ^ hasIPAQLevel(?e, "low") -> vectorItemExercise(?p, 2)	**2**

The selected educational material is an article titled “Jogging is economical and effective for lowing blood pressure”. The extracted 5 keywords of the article (using the improved TextRank algorithm) are “jogging”, “exercise”, “lowering blood pressure”, “hypertensive patient” and “running”. We will map the extracted keywords to the ontology vector space to generate the text vector. One thing to note is that “hypertensive patient” is a compound word and it will be segmented into the atom words (“hypertension” and “patient”) to perform the mapping. The mapping is

executed according to the cosine similarity between the word embeddings of the keyword and the vector item. If the cosine similarity is greater than 0.5, the cosine similarity value will be added to the corresponding vector item value. In this material, the mapping results are: “jogging” mapped to “exercise” (similarity: 0.67); “exercise” mapped to “exercise” (similarity: 1); “lowering blood pressure” mapped to “hypertension” (similarity: 0.55), “anti-hypertensive drug” (0.52) and “blood pressure” (0.50); “hypertension” mapped to “hypertension” (similarity: 1); “patient” mapped to nothing; “running” mapped to “exercise” (similarity: 0.71). Therefore, the non-zero items of the text vector are “exercise” (value: 2.38), “hypertension” (value: 1.55), “blood pressure” (value: 0.50) and “anti-hypertensive drug” (value: 0.52). The inner product between the patient vector and the text vector is 7.83, which achieves the top 1 recommendation among the 100 selected materials. The patient will receive this material on his/her smartphone through our mHealth system.
